# Photosensitive Hybrid γδ-T Exosomes for Targeted Cancer Photoimmunotherapy

**DOI:** 10.1021/acsnano.4c11024

**Published:** 2025-01-25

**Authors:** Yifan Gao, Jinzhao Liu, Meicen Wu, Yanmei Zhang, Manni Wang, Qingyang Lyu, Wenyue Zhang, Yang Zhou, Yin Celeste Cheuk, Xiwei Wang, Yinping Liu, Weiping Wang, Wenwei Tu

**Affiliations:** † Department of Paediatrics & Adolescent Medicine, Li Ka Shing Faculty of Medicine, The University of Hong Kong, Hong Kong SAR, China; ‡ State Key Laboratory of Pharmaceutical Biotechnology, The University of Hong Kong, Hong Kong SAR, China; § Department of Pharmacology and Pharmacy, Li Ka Shing Faculty of Medicine, The University of Hong Kong, Hong Kong SAR, China; ∥ Dr. Li Dak-Sum Research Centre, The University of Hong Kong, Hong Kong SAR, China

**Keywords:** γδ-T exosomes, photosensitive nanoparticles, hybrid exosomes, cancer immunotherapy, photoimmunotherapy

## Abstract

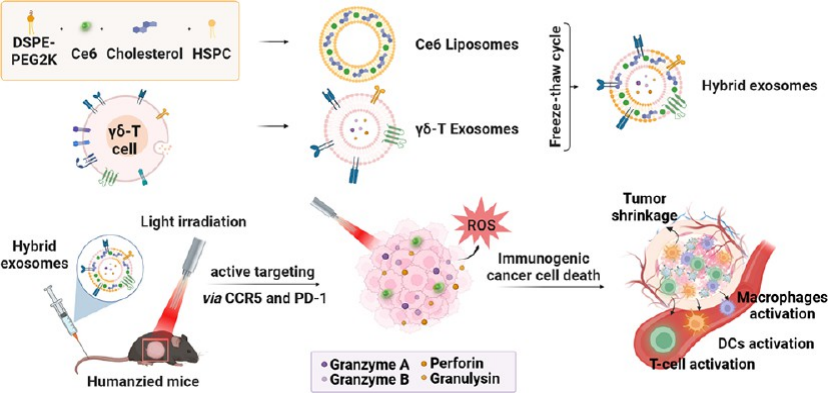

Melanoma is the most aggressive type of skin cancers. Traditional chemotherapy and radiotherapy have limited effectiveness and can lead to systemic side effects. Photodynamic therapy (PDT) is a photoresponsive cancer therapy based on photosensitizers to generate reactive oxygen species (ROS) to eradicate tumor cells. Our previous study showed that exosomes derived from human γδ-T cells (γδ-T exosomes) could control Epstein–Barr virus-associated tumors. Here, we combined γδ-T exosomes and PDT for targeted photoimmunotherapy by membrane fusion of γδ-T exosomes and Chlorin e6 (Ce6)-loaded liposomes. The functional surface proteins, such as CCR5 and PD-1, on the hybrid exosomes mediated the specific binding of hybrid exosomes toward melanoma tissues. The cytolytic molecules, such as granzyme A, granzyme B, perforin, and granulysin from γδ-T exosomes, induced specific apoptosis of cancer cells without harming normal cells. In response to light irradiation, ROS generation inside melanoma cells synergized with cytolytic molecules to induce apoptosis and promote immunogenic cancer cell death (ICD). The subsequently released damage-associated molecular patterns (DAMPs) could stimulate human dendritic cell maturation and induce melanoma antigen-specific CD4^+^ and CD8^+^ T-cell responses, thereby enhancing antitumor immunity. This study provides a promising strategy by combining γδ-T exosomes and PDT for photoimmunotherapy, thereby expanding the clinical applications of γδ-T exosome therapy for cancer patients.

## Introduction

Melanoma is one of the most aggressive types of skin cancer, with high rates of metastasis and poor prognosis.^1^ With very few standard treatment options, the median survival is around three to 11 months.^2^ Conventional therapies for melanoma treatment, such as chemotherapy and radiotherapy, lack specific tumor-targeting effects and can only provide short protection from relapse over time.^3−5^ As a result, researchers have focused on the development of targeted cancer therapy to improve the treatment and prognosis of melanoma.^6^

Photodynamic therapy (PDT) is a minimally invasive therapeutic strategy and is suitable for melanoma treatment with spatiotemporal control. It is initiated by the absorption of photon energy by a photosensitizer, which can be activated from the ground state to the excited singlet state, either decaying back to the ground state through fluorescence emission or undergoing intersystem crossing to the triplet state.^7^ By interacting with molecular oxygen and other endogenous substances, the triplet excited photosensitizer will produce reactive oxygen species (ROS), such as ^1^O_2_, H_2_O_2_, and O_2_^–^^8^. Further local laser irradiation to the tumor lesions will activate photosensitizers to eradicate tumor tissue via ROS generation without impacting normal tissue^9^ and to induce immunogenic cell death (ICD) for long-term antitumor immunity.^10,11^ Typically, PDT can promote the release of damage-associated molecular patterns (DAMPs) from tumor cells, which may further promote the maturation of antigen-presenting cells (APCs) to present tumor antigens and activate the antitumor T-cell response.^12^

Human γδ-T cells are a small subset of T lymphocytes, comprising 2–10% of T-cells in peripheral blood and lymphoid tissues.^13^ Depending on the δ chain difference in their T-cell receptors (TCRs), γδ-T cells can be further divided into Vδ1 and Vδ2 T-cells.^14^ Among all, Vγ9 Vδ2-T cells are the majority of γδ-T cells located in the peripheral blood and lymphoid tissues.^15,16^ They can be activated and expanded in an MHC-independent manner by phosphoantigens, which are nonpeptidic, small, phosphorylated intermediates of the mevalonate pathway.^17^ Our previous studies have shown that pamidronate (PAM), a pharmacological aminobisphosphonate used for osteoporosis treatment, can induce isopentenyl pyrophosphate (IPP) accumulation in mammalian cells, which further stimulates the proliferation and cytotoxic effector function of Vγ9 Vδ2-T (γδ-T) cells *in vitro* and *in vivo*.^18−21^ We further demonstrated that PAM can effectively treat Epstein–Barr virus-induced B cell cancer in a humanized mice model through selective activation of γδ-T cells *in vivo*.^22^ However, the therapeutic efficiency of PAM-expanded γδ-T cells for solid tumors is considerably limited due to the tumor immunosuppressive microenvironment and poor infiltration of γδ-T cells into solid tumors.^20,23−25^ This has inspired us to explore further cell-free therapy, i.e., extracellular vesicles derived from γδ-T cells, to expand their clinical applications and improve the therapeutic efficacy for solid cancer treatment.

Exosomes are small extracellular vesicles (20–200 nm) originating from endosomes that shuttle lipids, proteins, and nucleic acids between different cells.^26,27^ Unlike artificial nanoparticles, they naturally inherit functional molecules from parental cells and exhibit high biocompatibility and stability during blood circulation.^25,28−30^ Recently, immune cell-derived exosomes have been developed for cancer treatment, such as exosomes derived from neutrophils,^31^ macrophages,^32−34^ natural killer^35^ and dendritic cells,^36^ which exhibited great potential in cancer immunotherapy. Meanwhile, our recent studies further revealed that exosomes derived from human γδ-T cells (γδ-T exosomes) can control EBV-associated tumors.^37,38^ They can not only directly induce cancer cell apoptosis but also promote the antitumor αβ-T-cell response. More importantly, we further demonstrated that γδ-T exosomes could be used to generate tumor vaccines when they are conjugated with tumor-associated antigens.^39^ Compared with other exosome types, γδ-T exosomes offer unique advantages. They not only serve as efficient nanocarriers for drug delivery but also possess dual antitumor properties, targeting and eliminating tumor cells while boosting the antitumor T-cell immune response.^37−39^ In contrast, exosomes from DC and NK cells typically only have the ability to either enhance the antitumor T-cell response or kill tumor cells.^37^ The tumor-targeting capability of γδ-T exosomes makes them effective vehicles for delivering therapeutic agents directly to the tumor site, improving treatment efficacy, and reducing off-target effects. Moreover, the ease of producing allogeneic γδ-T exosomes at a large scale for cancer therapy sets them apart from exosomes derived from other immune cells, such as DC and NK cells. While the initial outcomes of γδ-T exosome-based therapy showed promise, our preliminary study uncovered the inefficacy of a single treatment with γδ-T exosomes in halting the progression of highly malignant cancers like melanoma *in vivo*. This discovery led us to formulate a more potent immunotherapy approach using γδ-T exosomes. Given the superficial characteristics of melanoma, we opted for PDT as an ideal candidate for combination therapy due to its ability to induce tumor cell apoptosis and trigger antitumor immune responses.

In this study, taking advantage of both PDT and γδ-T exosome-based therapy, we engineered γδ-T exosomes to synergize with Chlorine e6 (Ce6)-mediated PDT for better performance against melanoma. Ce6 is a red light (650 nm)-responsive photosensitizer approved by the U.S. Food and Drug Administration (FDA) with desired biocompatibility and suitable photosensitivity.^40^ Compared with deep-seated cancer types, red-light irradiation may easily penetrate skin tissues to reach melanoma lesions with minimal side effects.^41^ To efficiently encapsulate photosensitizers within exosomes, we fabricated photosensitive hybrid γδ-T exosomes composed of γδ-T exosomes and Ce6-loaded liposomes via a membrane fusion method. During the preparation process, surface ligands and cytolytic molecules from exosomes were preserved, and the photosensitizers were integrated into the hybrid formulation with high encapsulation efficiency. The hybrid exosomes shared the advantages of both liposomes and γδ-T exosomes with high stability and biocompatibility as well as an active targeting effect on melanoma lesions. Upon light irradiation, hybrid exosomes exhibited a synergistic antitumor effect from cytolytic molecules and ROS generation to induce photosensitive cancer cell apoptosis, which further enhanced the ICD effect within melanoma cells to promote the maturation of human monocyte-derived dendritic cells and the generation of antigen-specific T cells. To the best of our knowledge, this is the first time that exosomes derived from human immune cells were engineered with PDT to target synergistic cancer therapy. Our work provides a promising strategy through combining γδ-T exosomes and PDT for photoimmunotherapy, which may expand the applications of γδ-T exosome therapy in cancer treatment.

## Results and Discussion

### Fabrication of Photosensitive Hybrid γδ-T Exosomes via Membrane Fusion

**Figure 1:**
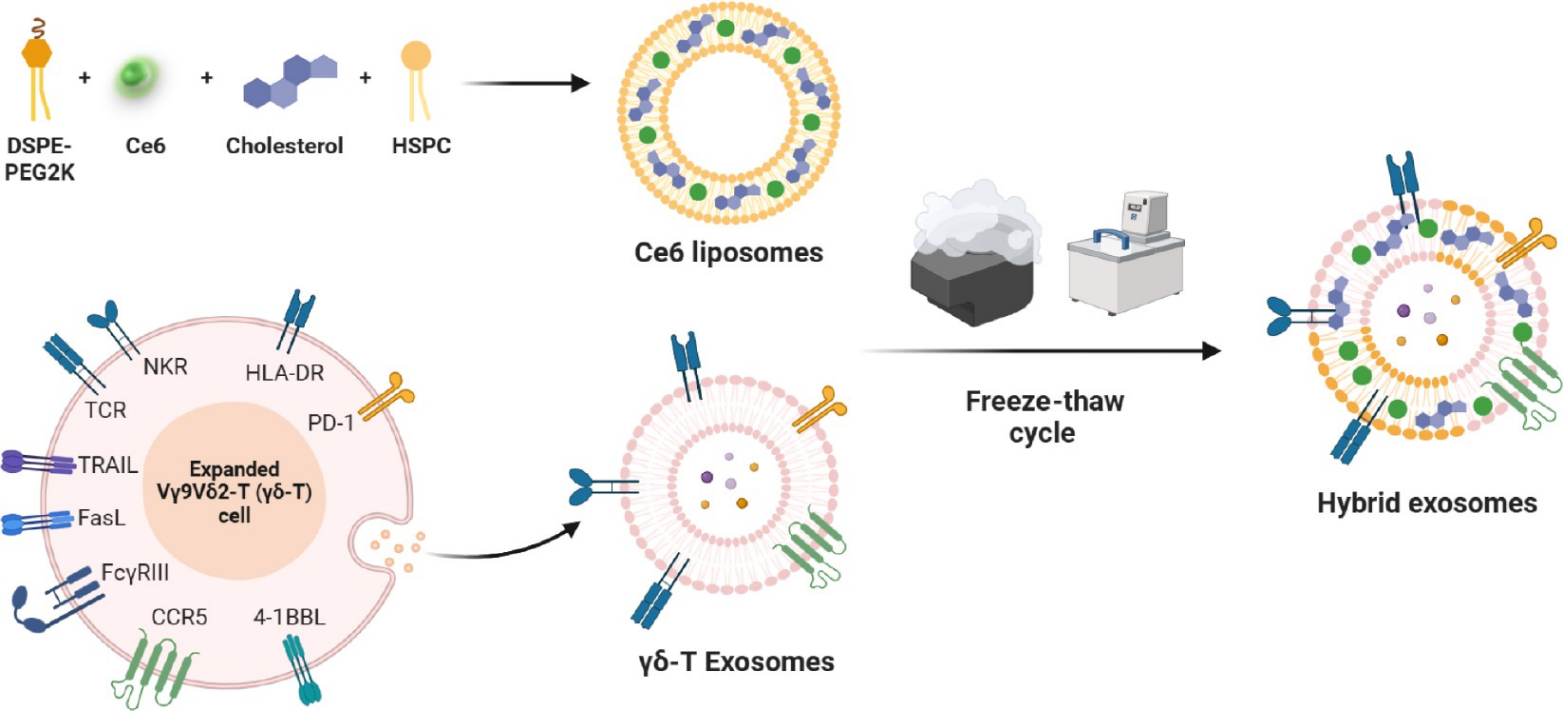
Schematic illustration of photosensitive γδ-T hybrid exosome preparation. Ce6 liposomes and Vδ2-T exosomes were premixed and then processed through three freezing-thaw cycles. The scheme was created on Biorender with publication license.

We first prepared the γδ-T exosomes and Ce6-loaded liposomes to fabricate photosensitive hybrid exosomes. γδ-T exosomes were collected through ultracentrifugation of the conditioned medium from PAM-expanded human γδ-T cells according to the protocol we reported before.^37^ The Ce6-loaded liposomes were prepared using the thin film hydration method.^42^ Previous studies have demonstrated that fusing liposomes to natural exosomes can be performed through various chemical and physical methods, including freeze–thaw fusion, natural incubation, polyethylene glycol-mediated fusion, and membrane extrusion.^43^ However, it has been reported that gentle conditions, such as natural incubation, will lead to a low loading efficiency, while intensive methods, such as membrane extrusion, will induce membrane disruption.^44^ In order to preserve the functional components of γδ-T exosomes to the maximum extent and balance cargo loading efficiency, we chose the freeze–thaw method. Other than permanent destruction, this method only generated temporary ice crystals in the freezing condition to disrupt the lipid bilayer structure of γδ-T exosomes for the fusion of the disrupted bilayer (Figure 1). As a result, the hybrid exosomes would retain both Ce6 and cytolytic molecules inside and functional markers on the surface.

**Figure 2:**
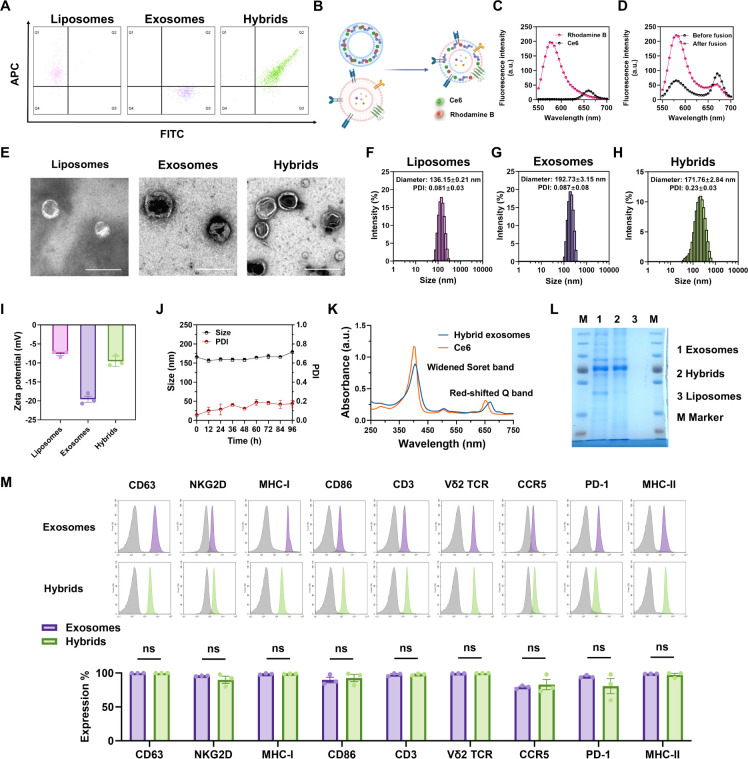
Preparation and characterization of photosensitive hybrid γδ-T exosomes. (A) Characterization of membrane fusion between liposomes and γδ-T exosomes by flowcytometric small particle detection. The representative FACS patterns for expression of Ce6 (APC channel) and γδ-T exosomes (FITC channel) in nanoparticles under the flowcytometric small particle detection were shown (*n* = 6). (B) Schematic illustration of the membrane fusion between liposomes and γδ-T exosomes and the increased distance between FRET pairs after fusion. (C) Fluorescence spectra of Rhodamine B and Ce6 (excitation at 540 nm, emission at 550–700 nm). (D) Fluorescence spectra change of hybrid γδ-T exosomes with Rhodamine B and Ce6 loaded before and after membrane fusion process (excitation at 540 nm, emission at 550–700 nm). (E) Transmission electron microscopy (TEM) images of Ce6-loaded liposomes, γδ-T exosomes, and hybrid γδ-T exosomes (scale bar: 200 nm). (F–H) Size distribution of Ce6-loaded liposomes, γδ-T exosomes, and hybrid γδ-T exosomes measured by dynamic light scattering (DLS). (I) Zeta potential of Ce6-loaded liposomes, γδ-T exosomes, and hybrid γδ-T exosomes measured by DLS. (J) Stability test of hybrid γδ-T exosomes under physiological conditions for 4 days (PBS, 37 °C). (K) UV–vis absorption spectra of free Ce6 and hybrid γδ-T exosomes in the presence of PBS buffer. (L) Protein composition of Ce6-loaded liposomes, γδ-T exosomes, and hybrid γδ-T exosomes by SDS-PAGE analysis. (M) Characterization of the surface markers of γδ-T exosomes and hybrid γδ-T exosomes by flow cytometry. Gray, stained with isotype control. Colors, stained with corresponding antibodies.

The successful fusion between γδ-T exosomes and liposomes was determined by small particle detection and the Förster resonance energy transfer (FRET) effect. Liposomes encapsulated with Ce6 were detected at APC channel^45^ and γδ-T exosomes stained with carboxyfluorescein succinimidyl ester (CFSE) were detected at the FITC channel. After freeze–thaw cycles, nanoparticles double positive for APC and FITC channels under the flow cytometric small particle detection were identified as hybrid exosomes (fusion efficiency: 94.40 ± 0.78 (*n* = 6)) (Figure 2A). The FRET phenomenon is another method to characterize the membrane fusion process.^46^ Since Rhodamine B and Ce6 fluorophores are suitable FRET donors and acceptors under 540 nm excitation, the liposomes encapsulating DSPE-Rhodamine B and Ce6 were prepared and then mixed with γδ-T exosomes (Figures S1 and 2B,C). After membrane fusion, diminished FRET activity was observed, possibly due to the increased distance between these two fluorophores (Figure 2D). The results suggested that the contents of γδ-T exosomes were inserted into the lipid bilayer of liposomes, indicating a successful membrane fusion process.

Transmission electron microscopy (TEM) images showed the intact cup-shaped morphology of hybrid exosomes with morphological properties of both Ce6-loaded liposomes and γδ-T exosomes (Figure 2E). Dynamic light scattering (DLS) demonstrated that Ce6-loaded liposomes and γδ-T exosomes had well-dispersed sizes of about 136.15 ± 0.21 nm and 192.73 ± 3.15 nm, respectively (Figure 2F,G). The size of hybrid exosomes was 171.76 ± 2.84 nm, and the polydispersity index (PDI) value was 0.23 ± 0.03, implying a suitable size distribution after membrane fusion (Figure 2H). The zeta potential of hybrid exosomes was −9.57 mV, which is a middle value between γδ-T exosomes and Ce6-loaded liposomes (Figure 2I). Encouragingly, the hybrid exosomes showed stable size distribution under physiological conditions for at least 96 h, suggesting their excellent stability during blood circulation (Figure 2J). For Ce6 drug loading during liposome preparation and membrane fusion, the encapsulation efficiency and loading capacity within hybrid exosomes were around 38% and 5%, respectively. The UV–vis absorption spectrum of hybrid exosomes exhibited a widened Soret band at 400 nm and a red-shifted Q-band at 650 nm compared with free Ce6, indicating the solid physical interactions of Ce6 and lipids and successful drug encapsulation (Figure 2K).

To identify protein components of hybrid exosomes, sodium dodecyl sulfate-polyacrylamide gel electrophoresis (SDS-PAGE) analysis showed that most proteins of the γδ-T exosomes were present in the hybrid exosomes (Figure 2L). In addition, we further identified the preservation of specific functional proteins on the surface of hybrid exosomes. As shown in Figure 2M, both hybrid exosomes and γδ-T exosomes displayed similar levels of CD63 (exosome marker), NKG2D, CD3, Vδ2 TCR, major histocompatibility complex-1 (MHC-I), CD86, programmed cell death protein 1 (PD-1), MHC-II, and C–C chemokine receptor type 5 (CCR5) on the surface, indicating surface markers of γδ-T exosomes were preserved from membrane fusion. To conclude, these results revealed that hybrid exosomes retained the properties of both γδ-T exosomes and Ce6-loaded liposomes after fabrication and might be endowed with active targeting and photosensitive apoptosis functions from two components.

### Hybrid γδ-T Exosomes Promoted Drug Accumulation within Human Melanoma Tissues

**Figure 3:**
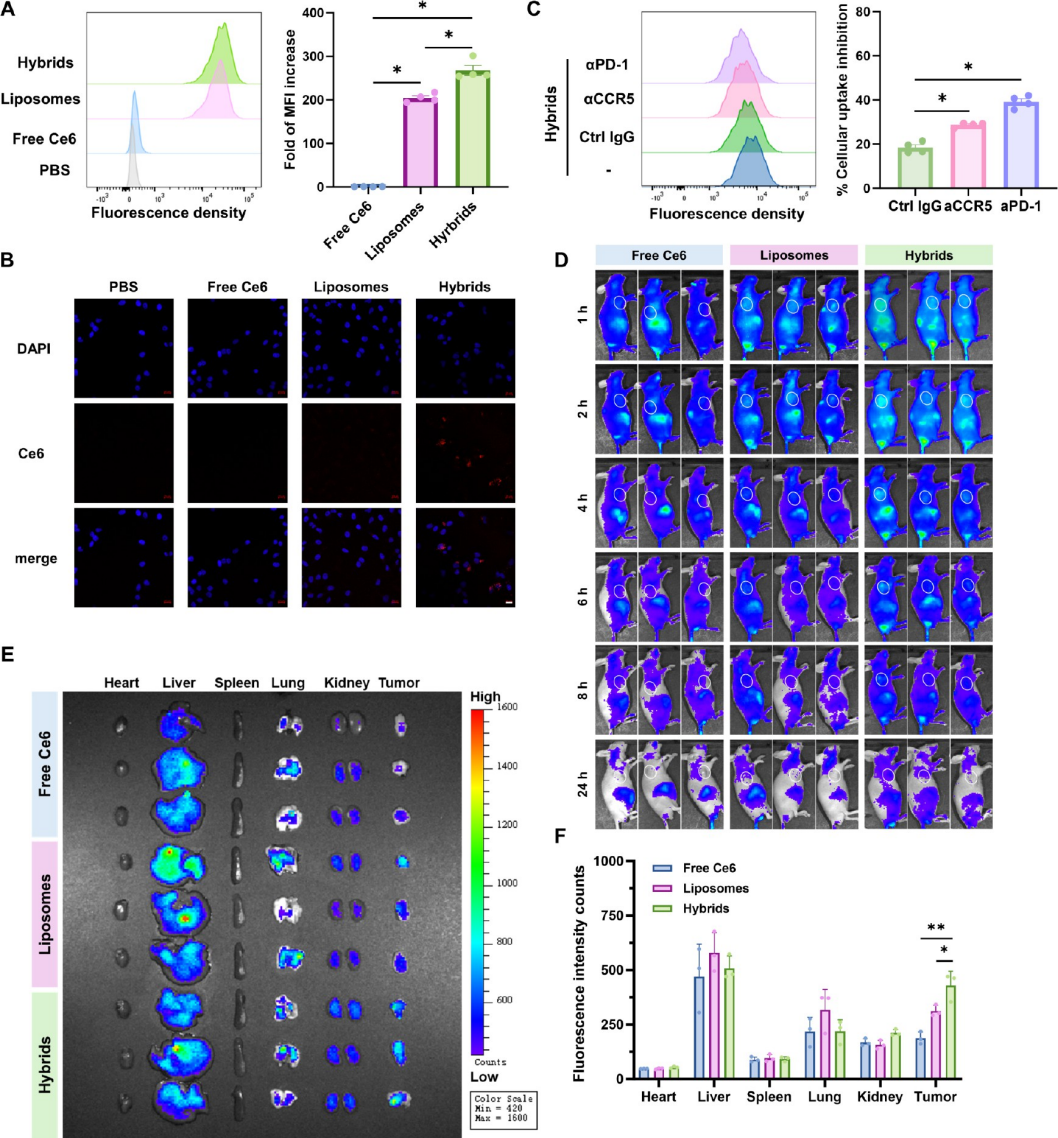
Active targeting effect of hybrid γδ-T exosomes to promote tumor accumulation. (A) Cellular uptake of Ce6 in A375 cells treated with PBS, free Ce6, Ce6-loaded liposomes, and hybrid γδ-T exosomes, measured by flow cytometry (*n* = 4). (B) Representative images of confocal laser scanning microscopy (CLSM) examination of Ce6 uptake inside A375 cells with different formulations (scale bar: 20 μm). (C) Flow cytometry measurement and quantification analysis of Ce6 cellular uptake levels in A375 cells treated with hybrid γδ-T exosomes with different functional antibodies (*n* = 4). (D) Fluorescence imaging and biodistribution of free Ce6, Ce6-loaded liposomes, and hybrid γδ-T exosomes in A375 tumor-bearing nude mice *in vivo* at different time points after injection. (E) Fluorescence imaging of tumor tissues and major organs *ex vivo* 24 h after injection. (F) Quantification results of fluorescent intensity of Ce6 in the tumor tissues and major organs *ex vivo* 24 h after injection (*n* = 3).

Given the targeting effect of γδ-T exosomes toward tumor cells demonstrated in our previous work,^37^ we hypothesized that hybrid exosomes also possess an active targeting effect on melanoma cells. First, we compared the cellular uptake level of Ce6 in melanoma cells *in vitro.* Human melanoma cells (A375) were treated with the same amount of Ce6 with different formulations (free drug, liposomes, or hybrid exosomes) for 4 h. Compared with Ce6-loaded liposomes, flow cytometric analysis showed that hybrid exosome treatment significantly enhanced the uptake level, suggesting hybrid exosomes had an advantage over liposomes in delivering drug molecules (Figure 3A). The same trend was also verified by confocal laser scanning microscopy (CLSM) imaging, with more red spots and stronger fluorescence in the cytoplasm of hybrid exosome-treated cells (Figure 3B). To investigate the underlying mechanism of tumor active targeting, we blocked the functional surface markers on hybrid exosomes and found that the increased cellular uptake level of hybrid exosomes was related to the surface expression of CCR5 and PD-1. When CCR5 or PD-1 was blocked by neutralizing αCCR5 or αPD-1 monoclonal antibody (mAb), the uptake level of hybrid exosomes in A375 cells was significantly decreased compared with that of the isotype IgG control (Figure 3C). These results indicated that functional markers on the surface of hybrid exosomes could mediate the active targeting effect and promote drug delivery toward melanoma cells.

To explore this active targeting effect of hybrid exosomes *in vivo*, an A375 xenograft tumor-bearing nude mice model was established. After intravenous injection of free Ce6, liposomes, and hybrid exosomes, the biodistribution of Ce6 was monitored by an *in vivo* imaging system (IVIS) at 650 nm excitation at different time points. The results demonstrated that hybrid exosomes accumulated significantly more than free drug and liposome groups within melanoma tissues throughout the observation (Figures 3D, S2). At 24 h post-administration, the mice were sacrificed, and tumor tissues and main organs were visualized under IVIS. For organ distribution, hybrid exosomes mainly accumulated in the liver and tumor tissues (Figure 3E). Regarding melanoma targeting, hybrid exosomes showed 2.29-fold and 1.37-fold more accumulation in tumor tissues compared with free Ce6 and liposome groups, respectively, indicating the advantages of hybrid exosomes in delivering drug molecules to tumor sites (Figure 3F). A similar trend was also confirmed by confocal analysis in tumor tissues (Figure S3). To conclude, both *in vitro* and *in vivo* experiments verified the active targeting effect of hybrid exosomes in melanoma tissues, possibly attributed to functional surface markers such as CCR5 and PD-1.

### Photosensitive Hybrid γδ-T Exosomes Induced Synergistic Cytotoxicity Effect Against Human Melanoma Cells

**Figure 4:**
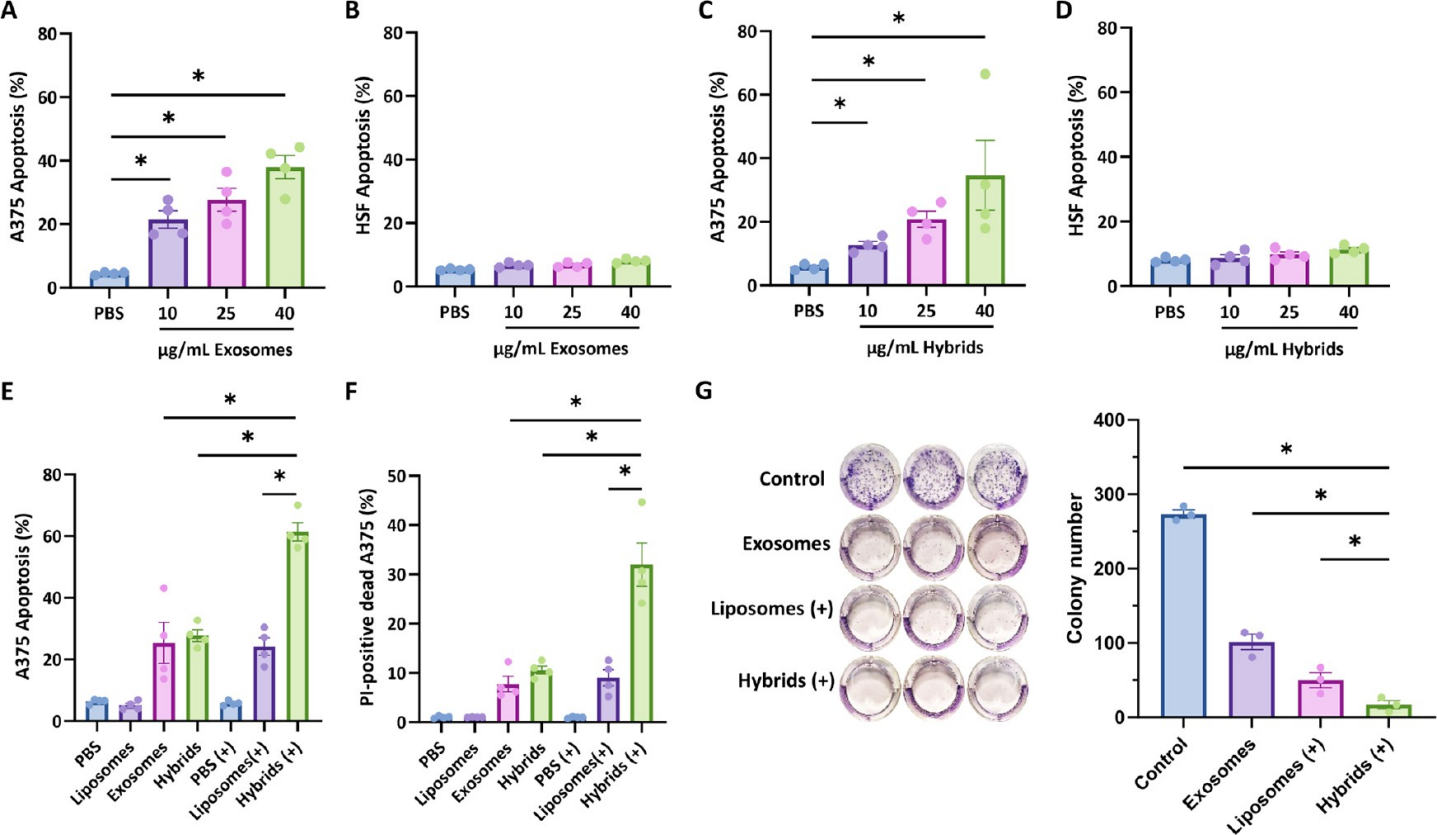
Photosensitive hybrid γδ-T exosomes induced synergistic cancer cell death. (A,B) The percentages of apoptotic A375 cancer cells or HSF skin fibroblasts after γδ-T exosomes treatment at different concentrations (*n* = 4). (C,D) The percentages of apoptotic A375 cancer cells or HSF skin fibroblasts after hybrid γδ-T exosomes treatments at different concentrations without light irradiation (*n* = 4). (E) The percentages of apoptotic A375 cells after different treatments with (+) or without light irradiation (*n* = 4). (F) Percentage of PI-positive dead A375 cells after different treatments with (+) or without light irradiation (*n* = 4). (G) Colony formation assay and quantification results of A375 cells after different treatment (*n* = 3). (+) means light irradiation, Xe lamp, 650 nm, 6.4 mW/cm^2^, 12 min.

To explore the antitumor effect of hybrid exosomes, we first examined the effect of γδ-T exosomes on human melanoma and normal cells. The results showed that human γδ-T exosomes could induce tumor cell apoptosis dose-dependently (Figures 4A). Notably, little apoptosis was detected when normal cells (human skin fibroblasts, HSF; HEK293T cells; human mesenchymal stem cells, MSCs) were treated with γδ-T exosomes at different concentrations (Figures 4B and S4), suggesting γδ-T exosomes possess specific cytotoxicity toward melanoma cells but not normal cells. Similarly, after hybrid exosome treatment without light irradiation, the apoptosis results also showed dose-dependent cytotoxicity toward tumor cells and high biocompatibility toward normal cells (Figure 4C,D). To investigate the combinational antitumor effect of γδ-T exosomes and PDT, A375 cells were incubated with liposomes, γδ-T exosomes, and hybrid exosomes with or without light irradiation (Figure 4E,F). In the dark condition, exosomes and hybrid exosomes showed equivalent cytotoxicity against A375 melanoma cells, indicating the percentages of apoptotic tumor cells and PI^+^ dead tumor cells. Upon red light irradiation (Xe Lamp, 650 nm, 6.4 mW/cm^2^, 12 min), liposomes led to a significant increase in cell apoptosis and cell death in A375 cells, which was comparable to that induced by exosomes without red light irradiation. Importantly, hybrid exosomes caused more than a 2-fold increase in melanoma cell apoptosis and death compared with γδ-T exosomes or liposomes with light irradiation (Figure 4E,F), indicating a strong synergistic antitumor effect of hybrid exosomes. The synergistic antitumor effect was further examined by colony formation assay. As shown in Figure 4G, hybrid exosomes with light irradiation exhibited a significantly stronger anticlonogenic effect than the other groups. These results demonstrated that hybrid exosomes possess a photoresponsive synergistic cytotoxic effect against melanoma cells.

### ROS Generation and Cytolytic Function of Photosensitive Hybrid γδ-T Exosomes

**Figure 5:**
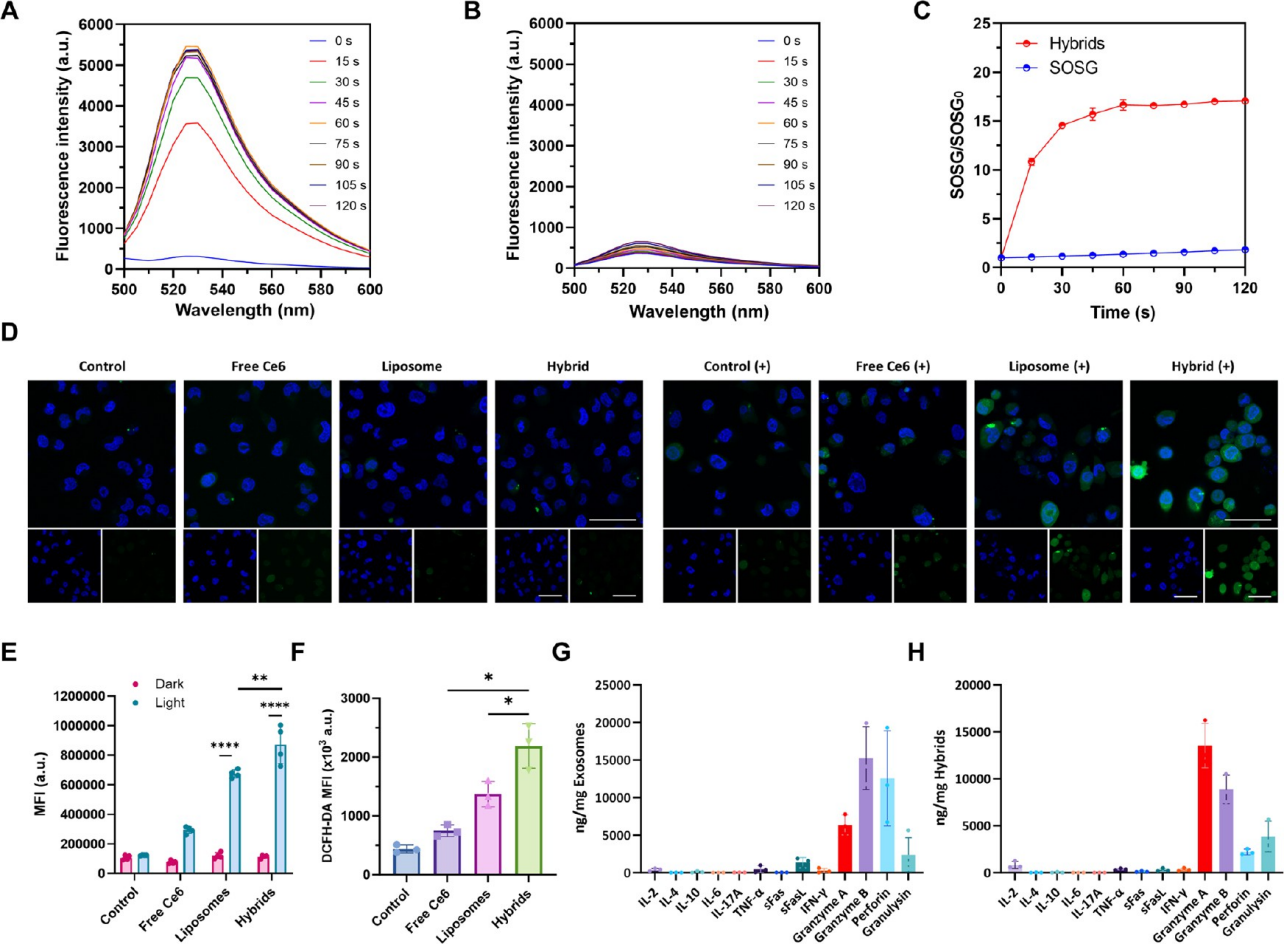
ROS generation ability and cytolytic function of photosensitive hybrid γδ-T exosomes. (A,B) Fluorescence spectra of hybrid γδ-T exosomes (A) or PBS buffer (B) with different light irradiation times using SOSG probe as the ^1^O_2_ sensor (excitation at 488 nm, emission at 500–600 nm). (C) Fluorescence changes at 525 nm of hybrid γδ-T exosomes with SOSG probe or SOSG probe alone with different light irradiation times. LED, 650 nm, 30 mW/cm^2^, 0–120 s. (D) Representative CLSM images of ROS generation inside A375 cells after different treatments with or without light irradiation (scale bar: 50 μm; blue: Hoechst 33342, green: DCFH-DA probe; Xe lamp, 650 nm, 6.4 mW/cm^2^, 12 min). (E) Quantification results of ROS generation in A375 cells after different treatments with or without light irradiation, measured by flow cytometry (*n* = 4, Xe lamp, 650 nm, 6.4 mW/cm^2^, 12 min). (F) Quantification results of ROS generation in tumor lesions in the A375 tumor-bearing nude mouse model (*n* = 3). (G,H) Analysis of cytolytic components within γδ-T exosomes or hybrid γδ-T exosomes, measured by LEGENDplex (*n* = 3).

To determine whether the photoresponsive cancer cell death of hybrid exosomes arises from PDT and ROS generation, we examined the singlet oxygen (^1^O_2_) generation by the SOSG probe. Upon red light irradiation, hybrid exosomes could produce a large amount of ^1^O_2_ in a time-dependent manner (Figure 5A–C). Inside A375 melanoma cells, the ROS level was measured with the help of the 2′,7′-dichlorodihydrofluorescein diacetate (DCFH-DA) probe under CLSM imaging. After light irradiation, hybrid exosomes could generate a much higher level of ROS in the cytoplasm and nucleus, in contrast to that in the dark (Figure 5D). Flow cytometric analysis showed similar results with significantly higher ROS production levels of irradiated hybrid exosomes compared with those of other groups (Figure 5E), which might be the underlying mechanism of hybrid exosome-mediated photoresponsive cancer cell apoptosis. *In vivo* ROS generation efficiency of PDT might be limited due to the hypoxic microenvironment in solid tumors.^47^ To confirm the ROS production *in vivo*, hybrid exosomes were intravenously injected into the A375 tumor-bearing nude mice. After light irradiation, the tumor tissues were collected and made into a single cell suspension for ROS detection. The results showed that hybrid exosomes could indeed increase the ROS level in the tumor tissues, which was significantly higher than those of the liposomes and free Ce6 groups (Figure 5F).

Apart from ROS generation, the other antitumor mechanism might come from the γδ-T exosomes. Therefore, we performed LEGENDplex to examine the level of cytolytic molecules in the γδ-T exosomes and hybrid exosomes. As shown in Figure 5G, γδ-T exosomes contained very high levels of cytolytic molecules such as granzyme A, granzyme B, perforin, and granulysin but only had a little of other cytokines, such as IL-2, IL-4, IL-10, IL-6, IL-17A, TNF-α, soluble FasL, and IFN-γ. Encouragingly, most of the cytolytic molecules such as granzyme A, granzyme B, perforin, and granulysin were still retained within hybrid exosomes after membrane fusion and nanoformulation fabrication (Figure 5H), which could explain their specific cytotoxicity to A375 cells without light irradiation. Further observation under a confocal microscope confirmed the successful entry of cytolytic molecules, such as granzyme B, into tumor cells (Figure S5). To conclude, the combinational strategy between γδ-T exosomes and PDT within hybrid exosomes mainly resulted from photoresponsive ROS generation and cytolytic molecules inherited from parental activated γδ-T cells, both of which contributed to the enhanced photosensitive antitumor effect on melanoma cells.

### Photoresponsive Hybrid Exosomes Triggered DAMPs Release and Induce Tumor Antigen-Specific αβ-T Cell Response

**Figure 6:**
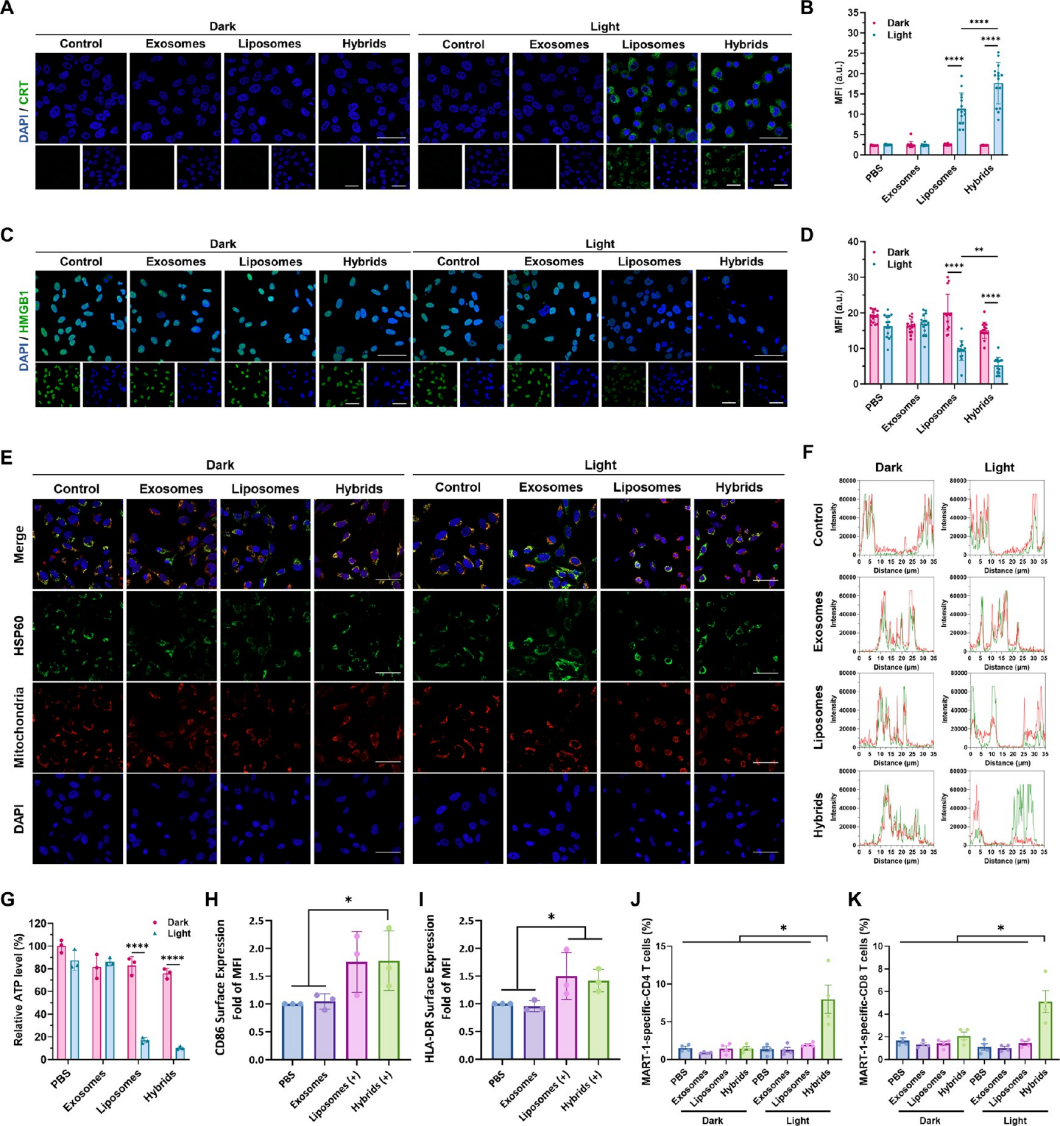
DAMPs release and dendritic cell maturation triggered by photosensitive hybrid γδ-T exosomes. (A,B) Representative CLSM images and quantification results of CRT exposure on the A375 cells after different treatments with or without light irradiation (scale bar: 50 μm; blue: DAPI, green: CRT). (C,D) Representative CLSM images and quantification results of HMGB1 release in the nucleus of A375 cells after different treatments with or without light irradiation (scale bar: 50 μm; blue: DAPI, green: HMGB1). (E) Representative CLSM images of colocalization between HSP60 and mitochondria inside A375 cells after different treatments with or without light irradiation (scale bar: 50 μm; blue: DAPI, green: HSP60, red: mitochondria). (F) Colocalization analysis between HSP60 and mitochondria inside A375 cells with different treatments (green line: HSP60; red line: mitochondria). (G) Relative ATP level inside A375 cells after different treatments with or without light irradiation (*n* = 3). (H,I) Flow cytometry analysis and quantification results of CD86 and HLA-DR on human dendritic cells (*n* = 3). Level of MART-1-specific CD4^+^ T-cells (J) and CD8^+^ T-cells (K) in different treatment groups. (*n* = 4). (+) means light irradiation, Xe lamp, 650 nm, 6.4 mW/cm^2^, 12 min.

It is widely reported that ROS generation triggered by PDT inside cancer cells will lead to endoplasmic reticulum (ER) stress-related immunogenic cell death (ICD).^48^ ICD is characterized by the upregulation of damage-associated molecular patterns (DAMPs), which can promote dendritic cell maturation, cytotoxic αβ-T cell activation, and systemic antitumor immune response.^49^ Since hybrid exosomes showed photosensitive ROS production and induced cancer cell apoptosis, we proposed that they could also induce the ICD effect in A375 cancer cells upon light irradiation. Representative CLSM images showed that calreticulin (CRT), an ER-residing chaperone protein in stressed cells,^50^ was significantly upregulated on the cell membrane of A375 cells after hybrid exosome treatment with light irradiation (Figure 6A,B). At the same time, high mobility group box 1 (HMGB1), a nuclear protein responsible for the maintenance of nucleosome structure,^51,52^ was released from the nucleus after hybrid exosome treatment with light irradiation (Figure 6C,D). Moreover, another DAMP signal was mitochondrial heat shock protein 60 (HSP60)^53^ which showed less colocalization with the mitochondria in the irradiated hybrid exosome group (Figure 6E,F). Lastly, a decreased intracellular adenosine triphosphate (ATP) level was also observed in the hybrid exosome group by a luminescence assay (Figure 6G). These results indicated the successful DAMP release and hybrid exosome-mediated ICD effect inside A375 cancer cells.

Besides DAMPs release, another consideration for cancer immunotherapy is dendritic cell maturation. Therefore, we cocultured the cancer cells stressed above with human monocyte-derived dendritic cells to examine the ICD effect. After 24 h, we found increased CD86 and HLA-DR expression in hybrid exosome and liposome groups, suggesting that hybrid exosome and liposome treatments could promote human dendritic cell maturation (Figure 6H,I). To explore the subsequent melanoma antigen-specific αβ-T-cell response, human PBMCs were cultured with the above-stressed tumor cells for 5 days and restimulated with a MART-1 peptide pool, covering the complete sequence of the human MelanA/MART-1 protein. As shown in Figure 6J,K, a significantly higher level of MART-1-specific CD4 and CD8 T-cell response in the photoirradiated hybrid group was observed. These results demonstrated that the photosensitive hybrid γδ-T exosomes could induce a light-triggered ICD effect, promote dendritic cell maturation, and induce a melanoma antigen-specific CD4 and CD8 T-cell response, and finally enhance antitumor immunity.

### *In Vivo* Antimelanoma Growth Effect of Photosensitive Hybrid γδ-T Exosomes

**Figure 7:**
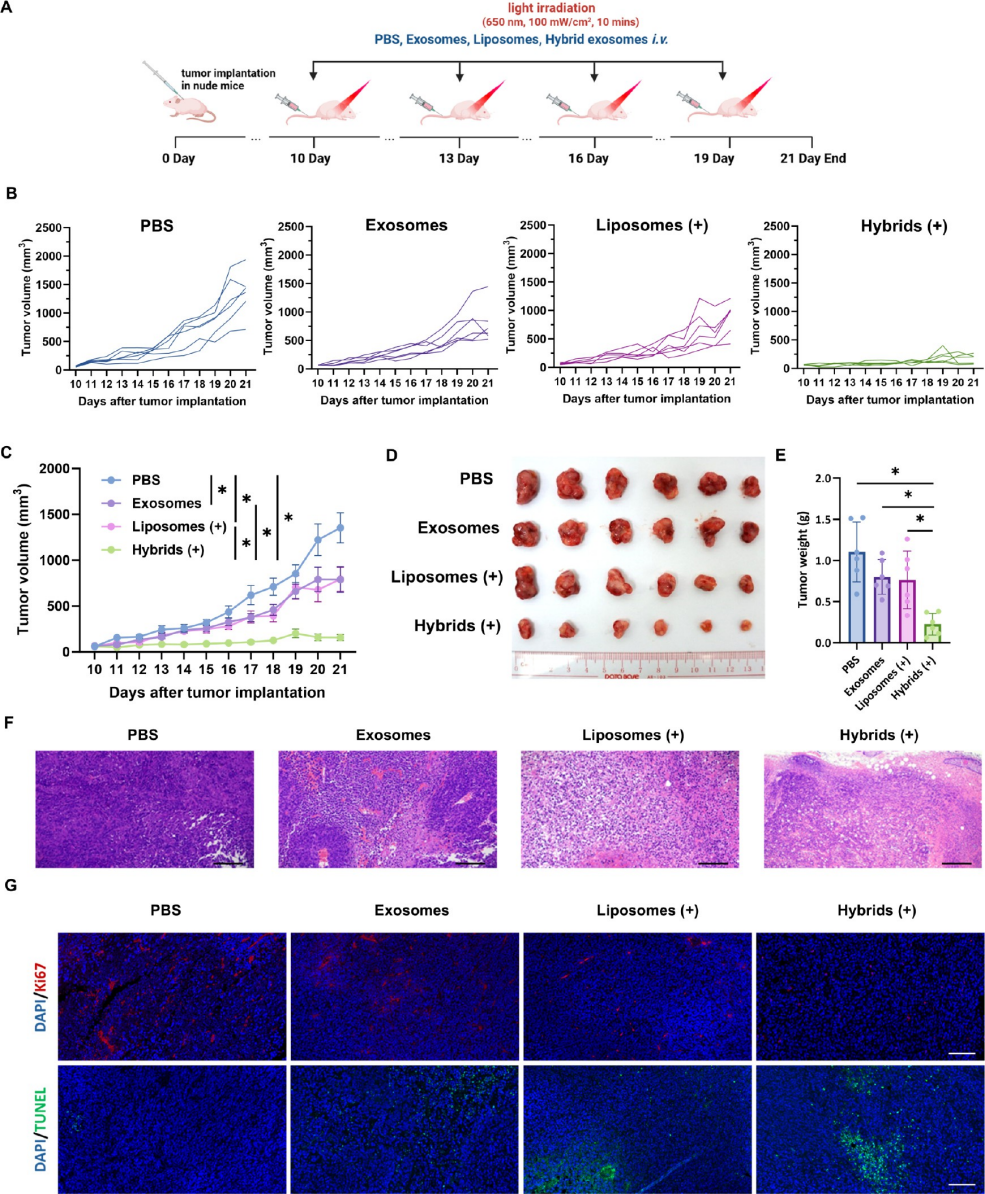
*In vivo* antimelanoma growth effect of photosensitive hybrid γδ-T exosomes in A375 tumor-bearing nude mice. (A) Schematic illustration of tumor implantation and treatment schedule during antitumor study. (B) The tumor volume growth curve of individual mice in four groups (*n* = 6 for each group). (C) Tumor volume growth curve of the mice with different treatments. (D) The image of tumors isolated from A375 tumor-bearing nude mice at the end of antitumor study. (E) Tumor weight of the mice with different treatments (*n* = 6 for each group). (F) Hematoxylin and eosin (H and E) staining of the tumor tissue sections from A375 tumor-bearing nude mice with different treatments (scale bar: 200 μm). (G) Representative CLSM images of Ki67 and TUNEL in the tumor tissue sections from A375 tumor-bearing nude mice after different treatments (scale bar: 100 μm). (+) means light irradiation, LED, 650 nm, 100 mW/cm^2^, 10 min.

To explore the antitumor effect of hybrid exosomes *in vivo*, the A375 xenograft tumor-bearing nude mice model was established. In brief, 1 × 10^5^ A375 cells were subcutaneously inoculated into nude mice 10 days before the treatment. Four groups of mice with different treatments were monitored during the therapeutic period. Different formulations were administered intravenously into the mice every 3 days from day 10 for up to 4 doses (Figure 7A). During the treatment, both γδ-T exosomes and Ce6-loaded liposome treatments showed a comparable antitumor effect to control melanoma tissue growth compared with the control group, as indicated by the changes in tumor volumes (Figure 7B,C). More importantly, hybrid exosomes exhibited a synergistic effect on suppressing tumor growth during treatment (Figure 7B,C). At the endpoint, tumor tissues were collected, imaged, and measured. As shown in Figure 7D,E, the treatment of γδ-T exosomes and Ce6-loaded liposomes resulted in a 28% and 32% tumor weight decrease compared with those of the control group. Again, hybrid exosome treatment also showed a synergistic antitumor effect, as evidenced by the significant decrease in tumor volume and weight compared to the other groups. In addition, histological analysis showed more necrosis in tumor tissue from the mice treated with hybrid exosomes than in the other treatment groups (Figure 7F). The immunophenotypic analysis further revealed that tumor tissues had more terminal deoxynucleotidyl transferase-mediated dUTP nick-end labeling (TUNEL)-positive cells but fewer Ki67-positive cells in mice treated with hybrid exosomes compared with other treatment groups (Figure 7G), indicating that the tumor cells in hybrid exosome-treated mice had more apoptotic cells and lower proliferative capacity than those in mice with other treatments.

The hemolysis assay evaluated the biosafety issues of photosensitive hybrid γδ-T exosomes. Hybrid exosomes had high biocompatibility with red blood cells during circulation (Figure S6). Moreover, histological analysis revealed that there was no significant structural and morphological change in the organs, such as the heart, liver, spleen, lung, and kidney, from the mice with different treatments (Figure S7). Besides, it was observed that the body weight among different groups of mice remained stable during the treatment period, implying that hybrid exosomes did not cause obvious systemic toxicity (Figure S8). There was no significant difference in the serum levels of alanine aminotransferase (ALT) and aspartate aminotransferase (AST) among the mice with different treatments (Figure S9), indicating that these formulations of exosomes and liposomes have excellent biocompatibility and no apparent side effects on mice.

### *In Vivo* Antitumor Immunity Induced by Photosensitive Hybrid γδ-T Exosomes

**Figure 8:**
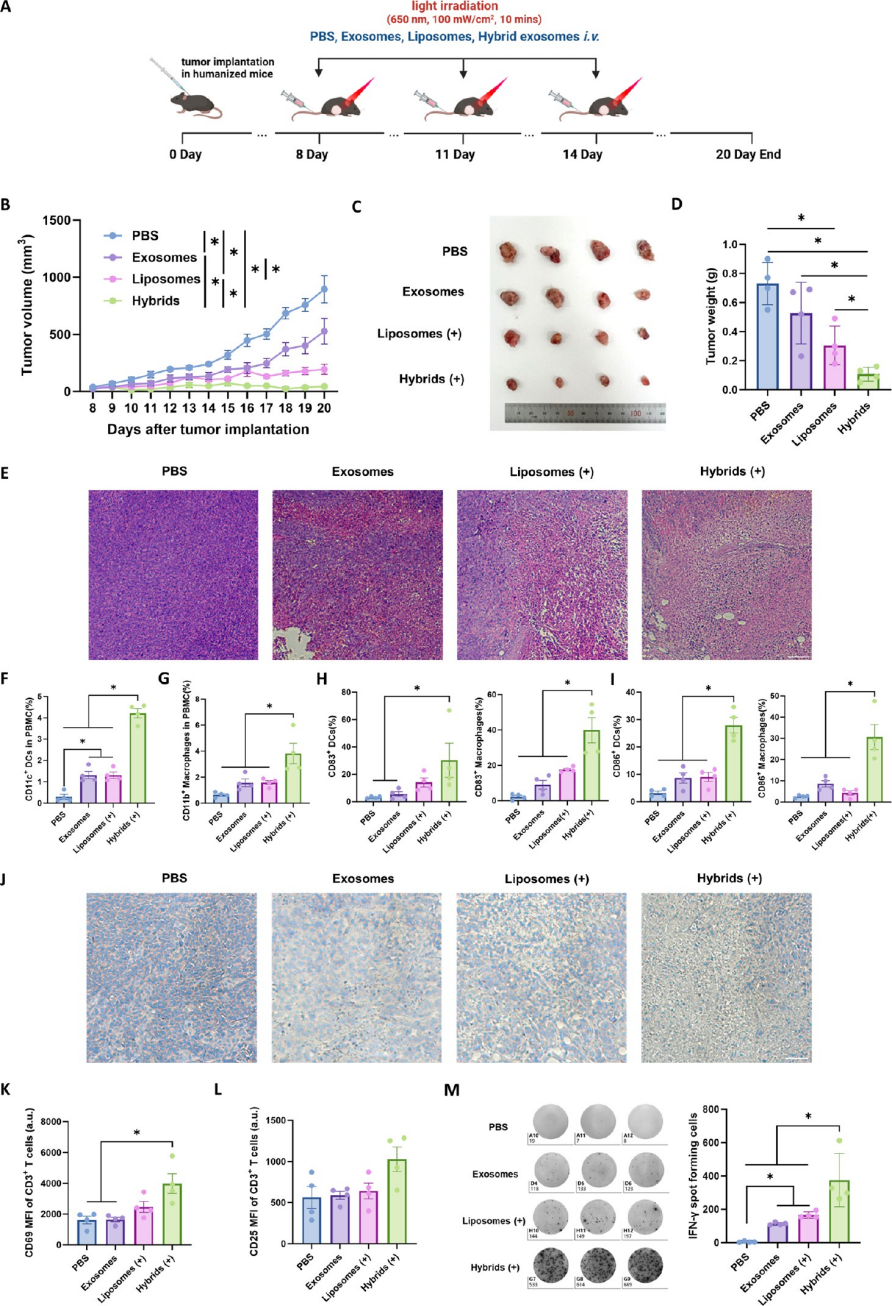
*In vivo* antitumor immune response induced by photosensitive hybrid exosomes. (A) Schematic illustration of tumor implantation and treatment schedule during antitumor study. (B) Tumor volume growth curve of the humanized mice with different treatments (*n* = 4 for each group). (C) The image of tumors isolated from A375 tumor-bearing humanized mice at the end of antitumor study. (D) Tumor weight in the humanized mice with different treatments (*n* = 4 for each group). (E) Hematoxylin and eosin (H and E) staining of the tumor tissue sections from humanized mice with different treatments (scale bar: 200 μm). The percentages of CD11c^+^ cells (F), CD11b^+^ cells (G), in PBMCs from humanized mice with different treatments. (H) Percentages of CD83^+^ cells in CD11c^+^ dendritic cells and CD11b^+^ macrophages in the peripheral blood from humanized mice with different treatments. (I) Percentages of CD86^+^ cells in CD11c^+^ dendritic cells and CD11b^+^ macrophages in the peripheral blood from humanized mice with different treatments. (J) Antihuman CD3 immunohistochemical staining (IHC) of the tumor tissue sections from humanized mice with different treatments (scale bar: 100 μm). (K) CD69 expression levels and (L) CD25 expression levels on CD3^+^ T-cells in the peripheral blood from humanized mice with different treatments. (M) Human IFN-γ-secreting cells detected by Elispot assay in the PBMCs from humanized mice with different treatments. (+) means light irradiation, LED, 650 nm, 100 mW/cm^2^, 10 min.

To determine the immune response induced by hybrid exosomes *in vivo*, humanized mice reconstituted with whole PBMCs were established as we described before.^21,22,54,55^ After 8 days of A375 tumor cell implantation, different treatments were given to humanized mice as illustrated in Figure 8A. Similar to that in nude mice, both γδ-T exosomes and Ce6-loaded liposome treatments could control melanoma tissue growth, and the hybrid exosomes exhibited a synergistic effect on suppressing tumor growth during the treatment in humanized mice (Figure 8B–D), indicating a therapeutic advantage of the hybrid exosomes over exosome or liposome monotherapy. Hematoxylin and eosin (H and E) staining of the tumor tissues also showed more necrosis of the tumor in hybrid exosome-treated humanized mice (Figure 8E).

At the endpoint of the animal study, human PBMCs were isolated from whole blood to analyze the immune response.^56,57^ As shown in Figure 8F,G, both γδ-T exosome and Ce6-loaded liposome treatments increased the frequencies of human monocyte-derived CD11c^+^ dendritic cells (DCs) and CD11b^+^ macrophages in the circulation, and hybrid exosomes had a synergistic effect on increasing the frequencies of these immune cells in circulation. Importantly, hybrid exosomes further induced the activation and maturation of these immune cells, as indicated by the increase in CD83 and CD86 expressions in these cells after hybrid exosome treatment (Figure 8H,I). These results demonstrated that hybrid exosomes could promote the function of APCs *in vivo*. Analysis of IHC staining in tumor tissues found that hybrid exosomes encouraged more human CD3^+^ T-cell infiltration into the tumor microenvironment (Figure 8J). In addition, hybrid exosomes also promoted the activation of peripheral blood CD3^+^ T cells, as indicated by the increases in CD69 and CD25 expressions on the surface of T-cells (Figure 8K,L). Further Elispot analysis for human IFN-γ-secreting cells in PBMCs confirmed the synergistic effect of hybrid exosomes on the IFN-γ production in human T-cells *in vivo* (Figure 8M). Taken together, our data from the humanized mice showed a consistent trend with the results obtained from the *in vitro* experiments, validating that hybrid exosome treatment could induce antitumor immunity *in vivo*.

## Discussion

In summary, we engineered photosensitive hybrid γδ-T exosomes by combining γδ-T exosomes with Ce6-loaded liposomes. This process efficiently encapsulated photosensitizers while preserving the essential functions of γδ-T exosomes. Our study demonstrated that the hybrid γδ-T exosomes selectively induced apoptosis of human A375 melanoma cells, triggered a synergistic antitumor effect through cytolytic molecules and ROS generation upon light irradiation, and initiated a strong ICD effect to promote dendritic maturation and induce melanoma antigen-specific T-cell response in both *in vitro* and *in vivo* settings. Importantly, the hybrid γδ-T exosomes inhibited melanoma growth in both nude and humanized mouse models without causing significant side effects to vital organs and tissues. This innovative hybrid exosome approach presents a synergistic strategy that combines γδ-T exosome therapy with PDT, offering a precise and efficient immunotherapeutic option for combating melanoma.

While previous studies have explored loading photosensitizers into exosomes for cancer therapy, our study marked the first time PDT was combined with exosomes derived from immune cells. Unlike conventional exosomes, such as HEK293 exosomes and tumor cell-derived exosomes, exosomes from immune cells contain essential outer and inner contents crucial for immunoregulation. Through a freeze–thaw membrane fusion method, we successfully loaded a photosensitizer into γδ-T exosomes using liposomes while preserving their antitumor functions. This pioneering approach may serve as a blueprint for future modifications of exosomes derived from various immune cells.

In recent years, there has been a surge in the exploration of human endogenous exosomes for targeted cancer therapy. These exosomes, derived from human cytolytic lymphocytes such as γδ-T exosomes and natural killer (NK) cells, exhibit cytotoxic activities against tumor cells.^58^ In addition, certain exosomes naturally carry therapeutic molecules, such as antitumor miRNA, that contribute to suppressing tumor growth.^59^ Despite the promising potential of exosomes in targeted cancer therapy, enhancing their inherent antitumor capabilities remains a significant challenge. In the current study, we pioneeringly engineered γδ-T exosomes to encapsulate a photosensitizer inside via membrane fusion with liposomes. Notably, this approach preserved the antitumor functions of γδ-T exosomes while achieving a high encapsulation efficiency of drug molecules. Our hybrid γδ-T exosomes demonstrated synergistic antitumor effects suitable for targeted photoimmunotherapy, offering a straightforward and stable nanoscale formulation for potential clinical applications.

Due to the leaky vasculature in the tumor microenvironment, nanoparticles can passively accumulate in tumor tissues based on the enhanced permeability and retention (EPR) effect. However, the EPR effect may vary among patients with different stages.^60^ Our results showed that, compared with liposomes, hybrid exosomes had an active targeting effect toward melanoma cells and led to higher drug accumulation levels within tumor tissues, which might be attributed to their surface functional markers, including PD-1 and CCR5, interacting with melanoma cells. In addition, the acidic condition in the tumor microenvironment, a hallmark of tumor malignancy, may also contribute to the accumulation of the hybrid exosomes in the tumor tissues in the mouse models because a low pH condition could improve the uptake of exosomes by tumor cells.^61^ The strategy can also be utilized to deliver other hydrophobic drug molecules and even hydrophilic cargos based on our hybrid γδ-T exosome formulation.

By combining γδ-T exosomes and PDT, we achieved a robust antitumor effect from γδ-T exosome-derived cytolytic molecules, including granzyme A, granzyme B, perforin, and granulysin, and Ce6-mediated ROS generation inside melanoma cells. Therefore, the hybrid exosome system is able to achieve a synergistic effect by combining the advantageous structures of γδ-T exosomes and Ce6 liposomes into a single nanoparticle system. In detail, the exosomal structure encouraged the Ce6 accumulation in melanoma cells, while the Ce6 liposomes enabled a synergistic photosensitive induction of tumor cell death with γδ-T exosomes. Consistent with reported PDT effects,^62^ the subsequent DAMPs release from light-irradiated tumor cells promoted dendritic cell maturation for antigen presentation and further induced tumor antigen-specific CD4^+^ and CD8^+^ T-cell responses, thus achieving photosensitive cancer immunotherapy.

The generation of a tumor antigen-specific T-cell response requires the maturation of dendritic cells and the presentation of tumor antigens to T-cells. Interestingly, although both Ce6 liposomes and hybrid exosomes could promote dendritic cell maturation, only light-irradiated hybrid exosomes induced robust tumor antigen-specific CD4^+^ and CD8^+^ T-cells *in vitro*. One possible explanation for this is that sufficient tumor antigens were released only in the hybrid group following large-scale tumor cell death, leading to an antigen-specific T-cell response. In our animal study, we confirmed that the hybrid exosomes effectively inhibited melanoma growth in the A375 tumor-bearing nude mice model, specifically inducing apoptosis and necrosis inside tumor tissues with light irradiation. In the humanized mice model, we further showed that hybrid exosomes could induce the maturation and activation of APCs and promote the activation and infiltration of T-cells for antitumor T-cell immunity.

The photosensitive hybrid γδ-T exosomes have several advantages over conventional chemotherapy and radiotherapy for the treatment of melanoma. First, the hybrid exosomes, by combining γδ-T exosomes with photosensitizers, can induce a synergistic antitumor effect, which enhances the efficacy of treatment compared with chemotherapy and radiotherapy, potentially leading to better outcomes for patients. Second, the hybrid exosomes have the capability to activate the immune system, promoting antitumor immune responses, which may help in the long-term control of cancer and reduce the risk of tumor recurrence. Third, the hybrid exosomes can actively target tumor cells, leading to the more precise delivery of therapeutic agents and their accumulation in cancer tissues. Finally, unlike conventional chemotherapy and radiotherapy, which can cause systemic toxicity due to nonspecific targeting, the targeted approach of hybrid exosomes may reduce collateral damage to healthy cells and tissues, minimizing side effects as confirmed in our animal studies.

In terms of the clinical translation feasibility of hybrid exosomes, γδ-T cells present a significant advantage in cancer immunotherapy due to their ease of *ex vivo* large-scale expansion. Established optimized protocols enable the expansion of homogeneous human γδ-T cells at a clinical scale, leading to the production of substantial quantities of γδ-T exosomes with sustained cytolytic activities against tumor cells and immunostimulatory properties.^63−65^ Pooling allogeneic γδ-T exosomes from numerous healthy individuals can enhance quality control, standardization, and centralization. Utilizing allogeneic γδ-T hybrid exosomes in cancer therapy may offer a more efficient and viable approach compared with autologous hybrid exosomes, demonstrating promise for future clinical translation.

## Conclusions

In conclusion, here we report the first bioengineering of human γδ-T exosomes and synergistic therapy between γδ-T exosome therapy and PDT. Our work introduces a promising therapeutic approach for efficiently and safely treating superficial carcinomas like melanoma. Furthermore, we outline a method for efficiently loading hydrophobic drug molecules into γδ-T exosomes while preserving their functions through the membrane fusion process. This study lays the groundwork for combining γδ-T exosomes with other antitumor treatments, such as chemotherapy and monoclonal antibodies, in synergistic therapy within biomimetic hybrid nanoparticle formulations. Apart from melanoma, our photoresponsive strategy might also be suitable to provide safe and efficient treatment for other high malignancy tumor types, such as retinoblastoma, colon cancer, breast cancer, etc. In summary, this project offers a promising approach by combining γδ-T exosomes with PDT for potent photoimmunotherapy and expands the potential clinical applications of γδ-T exosome therapy in cancer patients.

## Experimental Sections

### Study Design

This study aimed to determine the antitumor effects of hybrid γδ-T exosomes against melanoma. We first characterized the physicochemical properties of hybrid exosomes and profiled their biological profiles. Next, we explored the interaction of hybrid exosomes with melanoma tumor cells and normal skin fibroblasts. Furthermore, we evaluated the antitumor activity of the hybrid exosomes in the A375 tumor-bearing mouse models.

### Materials

Chlorin e6 (Ce6), Nile red, and crystal violet were purchased from Macklin (Shanghai, China). Hydrogenated soy phosphatidylcholine (HSPC), cholesterol, and *N*-(carbonyl-methoxypolyethylene glycol 2000)-1,2-distearoyl-*sn*-glycerol-3-phosphoethanolamine (DSPE-PEG2K) were purchased from AVT Pharmaceutical Tech Co., Ltd. (Shanghai, China). DSPE-Rhodamine B was purchased from Qiyuebio (Xi’an, China). SOSG probe, Calcein AM, Hoechst 33342, MitoTracker, and DAPI were obtained from Thermo Fisher Scientific (MA, USA). DCFH-DA was purchased from MedChemExpress (Shanghai, China). Propidium Iodide (PI) was obtained from Beyotime (Shanghai, China). Dimethyl sulfoxide (DMSO) was obtained from Sigma-Aldrich (Darmstadt, Germany). Acetonitrile (ACN), methanol, and other solvents were obtained from Oriental Co., Ltd. (Hong Kong, China). Antibodies used for immunofluorescence and flow cytometry and their origin were listed in Table S1.

### Cell Culture

Human melanoma cells A375 and human skin fibroblasts (HSF) were cultured in 10% fetal bovine serum (FBS)-Dulbecco’s modified Eagle medium (DMEM) at 37 °C in a 5% CO_2_ humidified atmosphere.

### Expansion of γδ-T Cells and Isolation of γδ-T Exosomes

Human PBMCs were isolated from buffy coats of healthy donors from the Hong Kong Red Cross through a Ficoll-Hypaque gradient centrifugation. Human PBMCs were expanded with PAM and human recombinant interleukin-2 (IL-2) as we described before.^22^ After 14 days of culture, expanded γδ-T cells were washed with PBS and cultured in exosome-free 10% FBS-RPMI 1640 medium with an IL-2 supplement. After 48 h, the conditioned medium was collected for exosome isolation. To remove dead cells and cell debris, the medium was centrifuged at 2000 g for 20 min at 4 °C and then centrifuged at 10,000 g for 30 min at 4 °C. After passing through a 0.22 μm syringe filter, the supernatant was centrifuged at 100,000 *g* for 70 min at 4 °C (Sw32Ti rotor, Beckman). Pellets were then resuspended in cold PBS (∼pH 7.4) for storage at −80 °C.^37^

### Fabrication of Ce6-Loaded Liposomes

Ce6-loaded liposomes composed of HSPC (54 mol %), cholesterol (30 mol %), DSPE-PEG2K (5 mol %), and Ce6 (11 mol %) were prepared by the thin film hydration method. The lipids and Ce6 were dissolved in a mixed solution of methanol and chloroform (3:1). The solution was evaporated to form a thin film on the bottom of the round-bottomed flask. Next, the thin film was hydrated with PBS buffer for 30 min at 56 °C to form the Ce6-loaded liposomes. To reduce the size, the liposomes were extruded against 400, 200, and 100 nm filters (Whatman) with an extruder (Avestin, LF-50). The unencapsulated Ce6 was removed by ultracentrifugation at 60,000 *g* for 1 h. To prepare FRET liposomes, DSPE-Rhodamine B with Ce6 was added to the methanol and chloroform (3:1) mix at the first step. The solution was evaporated, hydrated, and extruded, as described above.

### Fabrication of Photosensitive Hybrid Exosomes

Hybrid exosomes were prepared by membrane fusion. First, γδ-T exosomes and Ce6-loaded liposomes were mixed at a 1:1 (w/w) ratio and processed through freeze–thaw cycles. The fused nanoparticles were subjected to ultrafiltration to remove debris (Thermo Fisher Scientific).

### Small Particle Detection

To detect the fusion efficiency of hybrid exosomes, the Ce6-loaded liposomes were prepared, while the γδ-T exosomes were labeled with CFSE-FITC. After three freeze–thaw cycles, the mixture was diluted in the flow buffer and run under NovoCyte Quanteon (Agilent) following the manufacturer’s protocol. The fusion efficiency was determined by the percentage of APC and FITC double-positive population under flow cytometry.

### FRET Detection

FRET liposomes encapsulated with DSPE-Rhodamine B and Ce6 were first prepared and mixed with γδ-T exosomes. Before membrane fusion, the fluorescence spectrum of the mixture (excitation at 540 nm, emission at 550–700 nm) was measured by a multimode microplate reader. After three freeze–thaw cycles, the fluorescence spectrum was measured again to observe the change in the fluorescent spectrum.

### Hybrid γδ-T Exosomes Characterization

For transmission electron microscopy (TEM) imaging, Ce6-loaded liposomes, γδ-T exosomes, and hybrid exosomes were separately fixed with 2% paraformaldehyde and dropped on Formvar carbon-coated copper grids, which were then stained with 2% phosphotungstic acid for negative staining. The morphology of processed nanoparticle samples was imaged under a Philips CM100 Transmission Electron Microscope (Philips). The size distribution and zeta potential of Ce6-loaded liposomes, γδ-T exosomes, and hybrid exosomes were measured by a dynamic light scattering (DLS) instrument (ZS90, Malven Instruments, Southborough, MA, USA). The stability test of hybrid exosomes was determined by DLS in PBS buffer for 96 h at 37 °C. The UV–vis absorption spectra of free Ce6 and hybrid exosomes were measured by a multimode microplate reader in PBS buffer. The concentration of Ce6 loaded in the hybrid exosomes was determined by high-performance liquid chromatography (HPLC) to calculate the encapsulation efficiency and loading capacity.
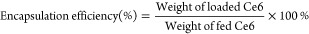

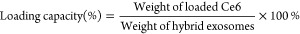


### Protein Profile and Surface Marker Analysis

For analysis of the protein profile of hybrid exosomes, the same amount of proteins within exosomes and hybrid exosomes were separated by electrophoresis on a 10% SDS-PAGE gel, which was then stained with Coomassie Blue Fast Staining Solution (Beyotime) for 1 h at room temperature and washed with ddH_2_O overnight. The image of the gel was captured under the Amersham Imager 680 Blot and Gel Imager (GE). Surface marker analysis of γδ-T exosomes and hybrid exosomes was conducted by incubating exosomes or hybrid exosomes with 4-μm aldehyde/sulfate latex beads (Thermo Fisher Scientific) overnight and blocking remaining binding sites. Then, the blocked beads were stained with fluorophore-conjugated flow antibodies against CD63, Vδ2 TCR, NKG2D, MHC-I, CD86, PD-1, MHC-II, and CCR5 or corresponding matched isotype controls (BioLegend). The surface expression of functional proteins was then analyzed under the NovoCyte Quanteon (Agilent).

### Cellular Uptake of Ce6 by A375 Cancer Cells

To compare the Ce6 uptake efficiency of free Ce6, Ce6-loaded liposomes, and hybrid exosomes, the same amount of Ce6 was administered to A375 cells. Four hours later, the uptake level of Ce6 was detected using the NovoCyte Quanteon (Agilent). For the confocal laser scanning microscopy (CLSM) assay, the A375 cells were washed with PBS three times and stained with DAPI for observation after different treatments. To analyze the mechanism of hybrid exosome-mediated active targeting, functional antibodies against PD-1, CCR5, or corresponding matched isotype controls were used in the coculture system. Four hours later, the uptake of Ce6 was compared using the NovoCyte Quanteon (Agilent).

### *In Vivo* Biodistribution and Tumor Accumulation

The Laboratory Animal Unit of the University of Hong Kong distributed BALB/c nude mice. All animals received care, and experiments were conducted according to the protocol approved by the Committee on the Use of Live Animals in Teaching and Research (CULATR) Li Ka Shing Faculty of Medicine (CULATR No. 5715–21, CULATR No. 22–172). Human melanoma cells A375 were inoculated subcutaneously into 4–6-week-old nude mice. The same amount of Ce6 in free form, liposomes, or hybrid exosomes was injected intravenously into the tumor-bearing nude mice. Whole-body images were captured at different time points after Ce6 systemic injection (*n* = 3) under a PE IVIS Spectrum. After 24 h, mice were sacrificed, and the fluorescence intensity of the tumor, heart, liver, spleen, lung, and kidney was compared under the PE IVIS Spectrum. For the tumor sectioning, nude mice bearing A375 tumors were treated with Ce6 in free form, liposomes, or hybrid exosomes intravenously. After 24 h, mice were sacrificed, and tumor lesions were collected for sectioning. The Ce6 accumulation within tumor tissues was observed under confocal laser scanning microscopy (CLSM).

### Annexin V/PI Apoptosis Assay

Human melanoma cells A375, or human normal skin fibroblasts (HSF), were treated with γδ-T exosomes, hybrid exosomes, or Ce6-loaded liposomes with or without light irradiation (Xe Lamp, 650 nm, 6.4 mW/cm^2^, 12 min). After 24 h, the cells were harvested and stained with FITC-Annexin V/PI in FITC-Annexin V binding buffer (BioLegend) at room temperature for 15 min. The stained cells were then analyzed under a NovoCyte Quanteon (Agilent) without washing.

### Colony Formation Assay

Human melanoma cells A375 were seeded in a six-well plate (800 cells per well) and treated with Vγδ-T exosomes, hybrid exosomes, or Ce6-loaded liposomes with or without light irradiation (Xe lamp, 650 nm, 6.4 mW/cm^2^, 12 min). After 7 days, cells were fixed with 4% PFA solution and stained with 0.1% crystal violet. After washing with PBS three times, A375 cells in each group were imaged.

### Singlet Oxygen (^1^O_2_) Detection

Hybrid exosomes were mixed with the SOSG probe (5 μM) in a PBS solution, and the fluorescence spectrum and 525 nm fluorescence were measured by the multimode microplate reader (excitation at 488 nm, emission at 500–600 nm) with different light irradiation times to detect singlet oxygen generation (LED, 650 nm, 30 mW/cm^2^, 0–120 s).

### ROS Generation Measured by CLSM or Flowcytometry

Human melanoma cells A375 were seeded in confocal dishes (Corning 200350) and treated with γδ-T exosomes, hybrid exosomes, or Ce6-loaded liposomes for 4 h. Then, the cells were treated with or without light irradiation (Xe lamp, 650 nm, 6.4 mW/cm^2^, 12 min). After PBS was washed three times, A375 cells were stained with DCFH-DA and Hoechst 33342 and observed by CLSM. For flow cytometry analysis, A375 cells after treatment were stained with DCFH-DA and analyzed under Novocyte Quanteon (Agilent). To measure the ROS generation level in tumor tissues, free Ce6, liposomes, or hybrid exosomes were intravenously injected into the A375 tumor-bearing nude mouse model. After 1 h, tumor tissues were irradiated for 10 min using red light LED (650 nm, 100 mW/cm^2^, 10 min). After another 30 min, the mice were sacrificed, and the tumor tissues were collected and made into a single-cell suspension. The ROS generation level was measured by flow cytometry using the DCFH-DA probe.

### Analysis of Cytolytic Components by LEGENDplex

γδ-T exosomes and hybrid exosomes were diluted in the assay buffer of the LEGENDplex Human CD8/NK Panel V02 (BioLegend). The samples were then prepared following the manufacturers’ protocols and analyzed on the BioLegend website .

### Immunofluorescent Staining of CRT and HMGB1

Human melanoma cells A375 were seeded in confocal dishes (Corning 200350) and treated with γδ-T exosomes, hybrid exosomes, or Ce6-loaded liposomes with or without light irradiation (Xe Lamp, 650 nm, 6.4 mW/cm^2^, 12 min). After 24 h, the cells were fixed with 4% PFA for 15 min at room temperature and washed three times with PBS. Subsequently, cells were permeabilized with 0.2% Triton X-100 and blocked in 3% BSA in PBS for 1 h at room temperature. Cells were incubated with anti-CRT or HMGB1 primary antibodies overnight at 4 °C. After being washed with PBS, the cells were stained with FITC-conjugated secondary antibodies for 1 h at room temperature. After being washed with PBS, the cells were stained with DAPI and mounted with antifade fluorescent medium (ab104135, Abcam). The quantification results were measured using ImageJ software.

### Colocalization Analysis of HSP60 and Mitochondria

Human melanoma cells A375 were seeded in confocal dishes (Corning 200350) and treated with γδ-T exosomes, hybrid exosomes, or Ce6-loaded liposomes with or without light irradiation (Xe lamp, 650 nm, 6.4 mW/cm^2^, 12 min). After 24 h, cells were fixed, permeabilized, blocked, and incubated with FITC anti-HSP60 antibodies and MitoTracker. After being washed with PBS, the cells were stained with DAPI and mounted with Anti-Fade Fluorescence medium (ab104135, Abcam). The colocalization assay between HSP60 and mitochondria was measured by ZEN version 3.3 software.

### Intracellular ATP Level

Human melanoma cells A375 were seeded in a 96-well plate and treated with γδ-T exosomes, hybrid exosomes, or Ce6-loaded liposomes, with or without light irradiation (Xe lamp, 650 nm, 6.4 mW/cm^2^, 12 min). After 24 h, the cells of each group were collected, and the intracellular ATP level was measured following the protocol provided in the kit (Beyotime S0026).

### Induction of Human Dendritic Cells from Human CD14^+^ Monocytes and Maturation

CD14^+^ monocytes were purified from human PBMCs using human CD14 microbeads (Miltenyi Biotec) following the manufacturer’s protocol. Then, the purified CD14^+^ cells were cultured with 10% human AB serum (ABS) RPMI1640 supplemented with 20 ng/mL Granulocyte-macrophage colony-stimulating factor (GM-CSF) and 50 ng/mL IL-4 for 7 days. The success of dendritic cell differentiation was determined by the frequency of CD11c^+^ cells using flow cytometry.^57,66^ To analyze dendritic cell maturation, a suspension of differentiated dendritic cells was added to the pretreated A375 cells and cocultured with the supplement of GM-CSF and IL-4 for 24 h. The levels of maturation markers CD86 and HLA-DR were detected using the Novocyte Quanteon (Agilent).

### Antigen-Specific T-Cell Response

Human PBMCs were cultured with the pretreated A375 cells directly after the light irradiation (Xe lamp, 650 nm, 6.4 mW/cm^2^, 12 min) for 5 days in 10% ABS RPMI1640. Then, nonadherent cells were suspended and seeded into a 96-well plate and treated with MART-1 peptide pool (PepTivator Melan-A/MART-1—premium grade, human, Miltenyi Biotec). Two hours later, Brefeldin A (BFA) was added to the culture system at a concentration of 10 μg/mL for another 6 h. The intracellular level of IFN-γ was determined by flow cytometry.

### Establishment and Treatment of Human Melanoma in Nude Mice

Balb/c nude mice were maintained in the Laboratory Animal Unit of the University of Hong Kong. All animals received care and experiments were conducted according to the protocol approved by the Committee on the Use of Live Animals in Teaching and Research (CULATR) Li Ka Shing Faculty of Medicine (CULATR No. 5715-21, CULATR No.22-172). 4–6-week-old nude mice were implanted subcutaneously with A375 (1 × 10^5^ per mouse) to establish the human melanoma model. Ten days after implantation, when the tumor volumes were around 100 mm^3^, the mice were intravenously injected with an equivalent volume of PBS, γδ-T exosomes, Ce6-loaded liposomes, or hybrid exosomes every 3 days for up to 4 doses. The protein amount of exosomes was the same as that of hybrid exosomes, while the Ce6 concentration of liposomes was the same as that of the hybrid exosomes. Light irradiation was given to the liposomes and hybrid exosomes groups 1 h after each injection (LED, 650 nm, 100 mW/cm^2^, 10 min). The tumor volume and body weight of the experimental mice were monitored every day. Mice bearing tumors with diameters reaching 17 mm or experiencing 20% body weight loss were euthanized according to the Laboratory Animal Unit of the University of Hong Kong regulations. On day 21, all mice were euthanized, and tumor tissues were weighed and saved together with other organs for histological and immunofluorescence staining analysis: length × [width]^2^ × 0.52 = tumor volume.

### Establishment and Treatment of Human Melanoma in Humanized Mice

Rag2^–/–^γc^–/–^ mice were maintained in the Laboratory Animal Unit of the University of Hong Kong. 4–8-week-old nude mice were treated with human PBMCs to establish a humanized mice model as previously described.^54^ After one month, stable humanized mice were implanted subcutaneously with A375 (3 × 10^5^ per mouse) to establish the human melanoma model. Eight days after implantation, when the tumor volumes were around 50 mm^3^, the mice were intravenously injected with an equivalent volume of PBS, γδ-T exosomes, Ce6-loaded liposomes, or hybrid exosomes every 3 days for up to 3 doses. The protein amount of exosomes was the same as that of hybrid exosomes, while the Ce6 concentration of liposomes was the same as that of hybrid exosomes. Light irradiation was given to the liposomes and hybrid exosomes groups 1 h after each injection (LED, 650 nm, 100 mW/cm^2^, 10 min). The tumor volume and body weight of experimental mice were monitored every day. Mice bearing tumors with diameters reaching 17 mm or experiencing 20% body weight loss were euthanized according to the Laboratory Animal Unit of the University of Hong Kong regulations. On Day 20, all mice were euthanized, and tumor tissues were weighed and saved together with other organs for histological and immunofluorescence staining analysis: length × [width]^2^ × 0.52 = tumor volume. The blood samples were collected for flow cytometry and ELISpot assay.

### Histological and Immunofluorescence Staining Analysis

Tumor tissues or main organs (heart, kidney, liver, lung, and spleen) were fixed with 10% formalin before embedding in paraffin for sectioning. Then, the sections were subjected to hematoxylin and eosin (H and E) or immunohistochemistry (IHC) staining, and the images were captured by ECLIPSE Ni–U (Nikon). For immunofluorescence staining, the tumor tissue sections were dewaxed in xylene and gradient concentrations of ethanol step by step. The citrate buffer was used for antigen retrieval. Sections were washed with PBS, permeabilized with 0.3% Triton X-100, and blocked in 10% BSA in PBS for 1 h at room temperature. Then, samples were incubated with PE anti-Ki67 antibodies overnight at 4 °C or TUNEL agents (Beyotime C1088) at 37 °C for 1 h. After being washed three times with PBS, samples were stained with DAPI for 10 min. Coverslips were mounted with Anti-Fade Fluorescence medium, and representative images were captured by CLSM.

### Hemolysis Assay

Blood from healthy mice was collected and centrifuged at 3000 rpm for 10 min to acquire blood plasma. After washing with PBS three times, the plasma was incubated with hybrid exosomes at gradient concentrations at 37 °C for 3 h. Then, the solution was centrifuged, and the plasma pellet was imaged.

### ALT and AST Assay

At the end of the antitumor study, the blood serum of different groups was collected, and mouse alanine aminotransferase (ALT) and aspartate transaminase (AST) were measured for the determination of liver function using the AST activity detection kit (BC1560, Solarbio) and the ALT activity detection kit (BC1550, Solarbio) according to the manufacturer’s instructions.

### Surface Staining Cells and Latex Beads

Surface staining of cells and latex beads was conducted using the following antibodies: anti-CD63 (H2C6), anti-TCR-γδ (B6), anti-MHC-I (W6/32), anti-MHC-II (Tü39), anti-CD86 (GL-1), anti-CCR5 (2D7), anti-NKG2D (1D11), and anti-PD-1 (RUO). All samples were detected using a Novocyte Quanteon instrument (Agilent) and analyzed with FlowJo software (TreeStar) and NovoExpress software (Agilent).

### Statistics

Statistical data are presented as means ± SEM. For the comparison between two groups, the Mann–Whitney *U* test was applied. For the comparison of two or more groups, one-way ANOVA (Bonferroni correction) was applied. For the analysis involving two or more variables, two-way ANOVA (Kaplan–Meier log-rank test) was applied.

## Data Availability

All the data supporting the findings of this study are available within the article and/or the Supporting Information online.

## Supporting Information Available

Antibodies used in this work for immunofluorescence and flow cytometry experiments (Table S1); fluorescence excitation spectrum of Ce6 (Figure S1); quantification results of Ce6 accumulation in tumor tissue areas of different treatments at different time points after injection (Figure S2); Ce6 accumulation of different formulations in the tumor sections harvested at 24 hours post treatment (Figure S3); apoptosis assay of γδ-T exosomes to HEK293T and human mesenchymal stem cells (huMSCs) with different concentrations at different time points (Figure S4); confocal images of A375 cells treated with CFSE-stained γδ-T exosomes and confocal images of A375 cells treated with γδ-T exosomes stained with AF647 antihuman Granzyme B (Figure S5); hemolysis assay of hybrid γδ-T exosomes with different concentrations (Figure S6); hematoxylin and eosin (H and E) staining of the main organ (heart, kidney, liver, lung, and spleen) sections from A375 tumor-bearing nude mice with different treatments (Figure S7); body weight change of A375 tumor-bearing nude mice with different treatments (Figure S8); serum levels of ALT (alanine aminotransferase) and AST (aspartate aminotransferase) of A375 tumor-bearing nude mice with different treatments (Figure S9) (PDF)
