# Supplementary material for: Photosensitive Hybrid γδ-T Exosomes for Targeted Cancer Photoimmunotherapy
Source: ACS Nano. 2025 Jan 25;19(4):4251–68. doi: 10.1021/acsnano.4c11024 (PMC11803918; doi:10.1021/acsnano.4c11024)
Supplement: Supplementary file 1 — nn4c11024_si_001.pdf [file nn4c11024_si_001.pdf]

**Supplementary information**

# Photosensitive hybrid γδ-T exosomes for targeted cancer photo-immunotherapy

Yifan Gao[1] †, Jinzhao Liu[2,3,4] †, Meicen Wu[2,3,4], Yanmei Zhang[1], Manni Wang[1], Qingyang Lyu[2,3,4], Wenyue Zhang[1], Yang Zhou[2,3,4], Yin Celeste Cheuk[1], Xiwei Wang[1], Yinping Liu[1], Weiping Wang[2,3,4] *, Wenwei Tu[1] *

[1] Department of Paediatrics & Adolescent Medicine, Li Ka Shing Faculty of Medicine, The University of Hong Kong, Hong Kong SAR, China.

[2] State Key Laboratory of Pharmaceutical Biotechnology, The University of Hong Kong, Hong Kong SAR, China

[3] Department of Pharmacology and Pharmacy, Li Ka Shing Faculty of Medicine, The University of Hong Kong, Hong Kong SAR, China

[4] Dr. Li Dak-Sum Research Centre, The University of Hong Kong, Hong Kong SAR, China

*Corresponding author email: wwtu@hku.hk (Wenwei Tu), wangwp@hku.hk (Weiping Wang)

† These authors contributed equally.

## Content

1. TableS1...........................................................................................................p3

2. Fig. S1-S9..................................................................................................p4-p8

**Table S1:** Antibodies used in this work for immunofluorescence and flow cytometry experiments.

| Name | Cat # | Fluorophore | Company |
|---|---|---|---|
| Anti-human Calreticulin (CRT) antibody | A1066 | N/A | ABclonal |
| Anti-human HMGB1 antibody | A19529 | N/A | ABclonal |
| Goat Anti-Rabbit IgG H&L | ab6717 | FITC | Abcam |
| Anti-human HSP60 antibody | ab46798 | FITC | Abcam |
| Anti-human CD86 antibody | 374216 | PerCP/Cyanine5.5 | Biolegend |
| Anti-human CD63 antibody | 353006 | FITC | Biolegend |
| Anti-human NKG2D antibody | 320820 | FITC | Biolegend |
| Anti-human HLA-A,B,C | 311406 | PE | Biolegend |
| FITC Mouse IgG1, κ Isotype Ctrl | 981802 | FITC | Biolegend |
| PE Mouse IgG2a, κ Isotype Ctrl | 981910 | PE | Biolegend |
| PerCP/Cyanine5.5 Mouse IgG1, κ Isotype Ctrl | 981820 | PerCP/Cyanine5.5 | Biolegend |
| Anti-human CD3 Antibody | 344816 | PE/Cyanine7 | Biolegend |
| PE/Cyanine7 Mouse IgG1, κ Isotype Ctrl | 981816 | PE/Cyanine7 | Biolegend |
| Anti-human TCR Vδ2 Antibody | 331418 | APC | Biolegend |
| APC Mouse IgG1, κ Isotype Ctrl Antibody | 400120 | APC | Biolegend |
| Anti-human CD195 (CCR5) Antibody | 555992 | FITC | BD |
| FITC Mouse IgG2a, κ Isotype Control | 349051 | FITC | BD |
| Anti -human CD279 (PD-1) Antibody | 329918 | PE/Cyanine7 | Biolegend |
| Anti-HLA-DR antibody | 307603 | FITC | Biolegend |
| FITC Mouse IgG2a, κ Isotype Ctrl Antibody | 400208 | FITC | Biolegend |
| Anti-Ki67 antibody | 350503 | PE | Biolegend |

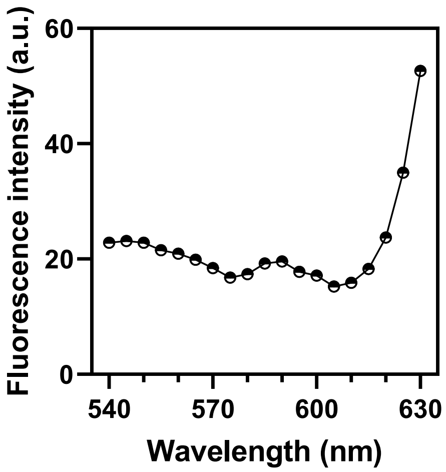


**Figure S1.** Fluorescence excitation spectrum of Ce6 (Excitation at 540-630 nm, Emission at 660 nm)

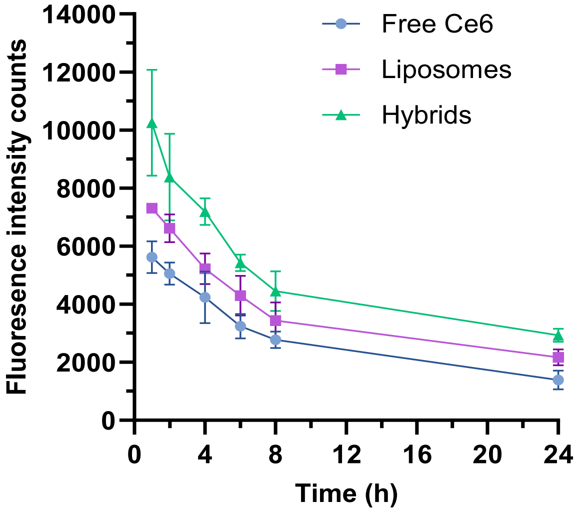


**Figure S2.** Quantification results of Ce6 accumulation at tumor tissue area of different treatments at different time points after injection (n = 3).

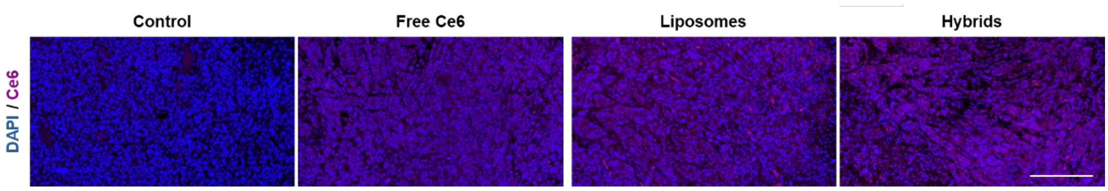


**Figure S3.** Ce6 accumulation of different formulations in the tumor sections harvested at 24h post treatment (Scale bar: 200 μm).

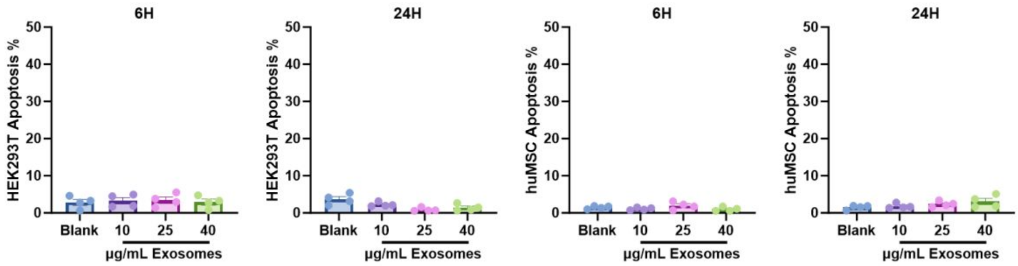


**Figure S4.** Apoptosis assay of γδ-T exosomes to HEK293T and human mesenchymal stem cells (huMSCs) with different concentrations at different timepoints (6 hours and 24 hours) (n = 4).

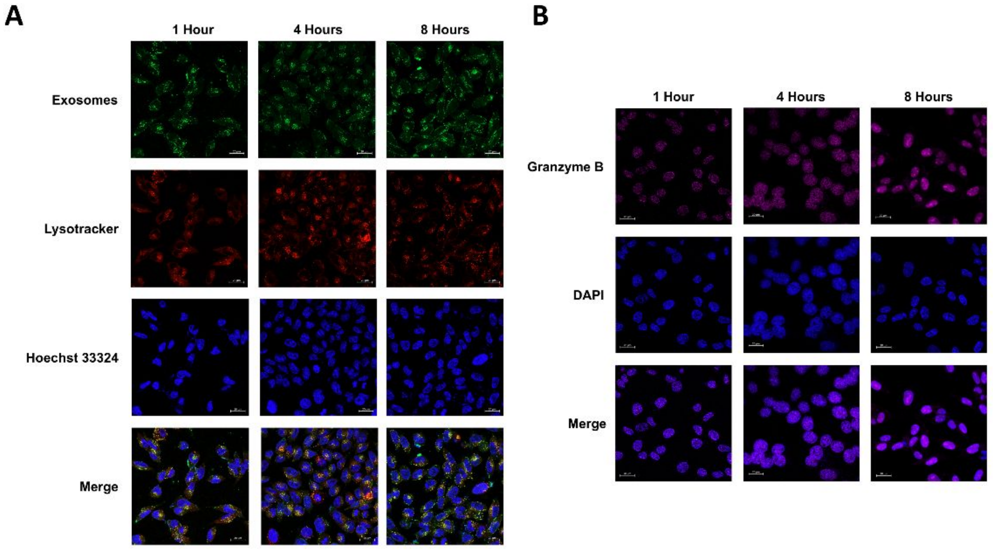


**Figure S5.** (A) Confocal images of A375 cells treated with CFSE staining γδ-T exosomes (Scale bar: 20 μm). Green, CFSE exosomes; Red, Lysotracker; Blue, Hoechst 33324. (B) Confocal images of A375 cells treated with γδ-T exosomes stained with AF647 anti-human Granzyme B (Scale bar: 20 μm). Pink, Granzyme B; Blue, DAPI.

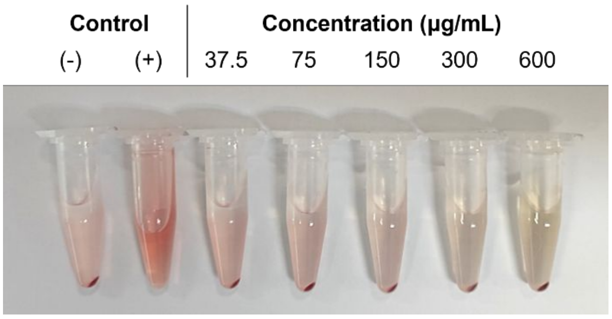


**Figure S6:** Hemolysis assay of hybrid γδ-T exosomes with different concentrations. (-) means negative control using PBS buffer. (+) means positive control using 1% Triton X-100 PBS buffer.

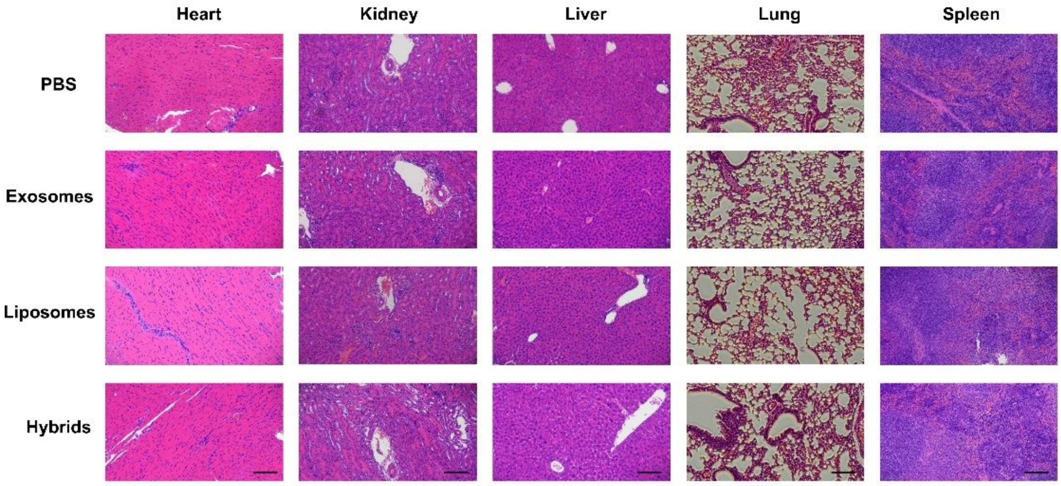


**Figure S7:** Hematoxylin and eosin (H&E) staining of the main organ (heart, kidney, liver, lung, and spleen) sections from A375 tumor-bearing nude mice with different treatments (Scale bar:200 μm).

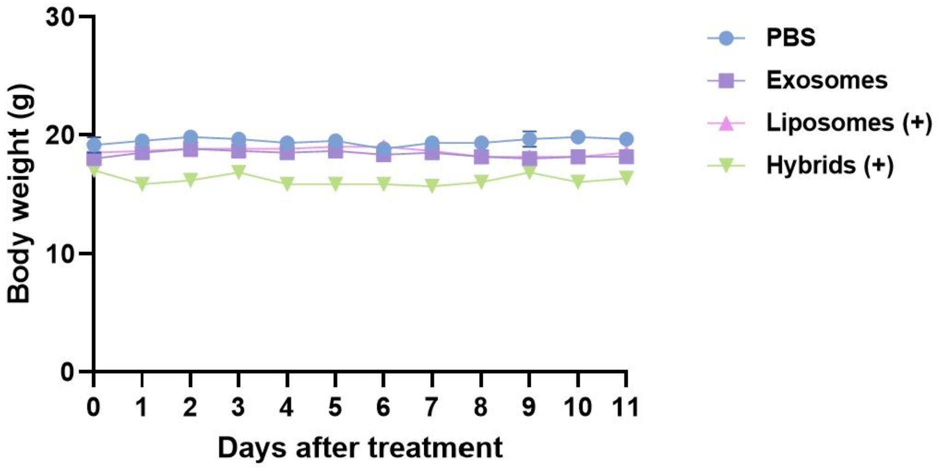


**Figure S8:** Body weight change of A375 tumor-bearing nude mice with different treatments (n = 6).

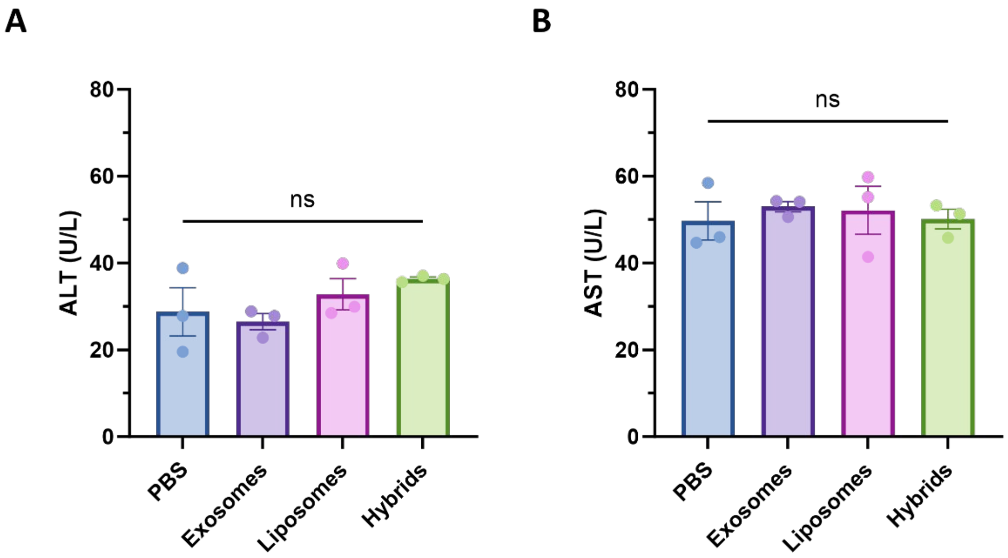


**Figure S9:** Serum levels of ALT (alanine aminotransferase) and AST (aspartate aminotransferase) of A375 tumor-bearing nude mice with different treatments (n= 3).
